# Inclusion and exclusion in gaming communities: a systematic review and future directions to increase inclusivity

**DOI:** 10.3389/fpsyg.2026.1796305

**Published:** 2026-05-01

**Authors:** Yiftach Argaman, Tamar Gur, Ben Heller, Yossi Maaravi

**Affiliations:** 1 Institut für Psychologie, University of Greifswald, Greifswald, Germany; 2 Adelson School of Entrepreneurship, Reichman University, Herzliya, Israel; 3 Baruch Ivcher School of Psychology, Reichman University, Herzliya, Israel

**Keywords:** community, diversity, exclusion, gaming, inclusion, marginalization, stereotypes

## Abstract

**Introduction:**

Gaming engages more than 3.6 billion youth and adults worldwide. Gaming in its current form facilitates rich social exchanges through diverse platforms, transforming how participants communicate, collaborate, and build relationships. Yet despite the theoretical accessibility of gaming spaces to individuals from all backgrounds, gaming communities frequently manifest high rates of exclusionary practices. The high rates of social exclusion undermine gaming’s promise as an inclusive domain, with participants from marginalized groups systematically being held back from full engagement in gaming.

**Methods:**

This paper systematically analyzed inclusivity challenges based on findings from 100 articles published over the last thirteen years.

**Results and Discussion:**

Our methodical examination of this literature identifies patterns of exclusion operating through representations, community practices, and institutional structures. We also document current inclusivity initiatives undertaken by key stakeholders, including developers, community organizers, and governing bodies, and develop new ones into an integrated framework to minimize exclusivity within gaming cultures. We argue that academic research can play a vital role in this transformation through facilitating cross-sector collaboration, evaluating intervention effectiveness, tracking emerging challenges, gathering participant perspectives, and disseminating practical knowledge. By offering interdisciplinary recommendations, this paper aims to outline a model for more equitable gaming communities that can fulfill their potential as sites of social inclusion.

## 1 Introduction

In our dynamic and increasingly digital world, gaming (specifically video games played on computers, consoles, or phones) has become much more than a source of entertainment; it has become a widespread cultural phenomenon. Once a hobby associated with a small demographic, the number of active gamers worldwide is now estimated at around 3.6 billion, projected to increase to over 3.9 billion by 2028 (Newzoo, 2025).

Due to the social elements intrinsic to games, many distinct and unique virtual communities have been established on social media and third-party platforms (e.g., Reddit, Discord), each with its own social trends and norms (Daneels et al., 2021; Gray and Leonard, 2018). The increases in the size of the player base and the opportunities provided by technological social platforms provide an ideal ground for inclusivity and diversification processes within these gaming communities. Yet, as many studies have shown, this potential is undermined by the reality of exclusionary and discriminatory communities and their norms (Banerjee et al., 2021; Gray and Leonard, 2018; Hayday and Collison, 2020; Vajawat et al., 2021; Waechter and Meschik, 2023). The current review aims to explore the varied barriers to inclusivity and diversity in gaming communities by examining the roles of the industry (e.g., investors, leaders, game designers), community members (e.g., other players, influencers), and the broader social and political ecosystem (e.g., regulators, non-governmental organizations). This review also goes a step further and aims to provide solutions to these obstacles, for each of the stakeholders involved.

### 1.1 The rise of gaming as a widespread phenomenon

Historically, games have long been part of human culture, due to their inherent drive for challenge, narrative, and social bonding (Peng et al., 2012; Wu et al., 2017). Naturally, advances in science and technology were quickly integrated into the gaming sphere, turning physical real-world games into arcade, console, personal computer, and most recently, virtual reality games (Allen and Anderson, 2018; Rieger et al., 2014). Video games are so ubiquitous in social spaces that even educational institutions, such as schools and libraries, have begun to incorporate them into their ecosystems (Snyder Broussard, 2012).

As previously mentioned, the gaming community today includes about 2.58 billion players worldwide (Statista, 2024), with approximately 85% of children aged 12 to 17 actively playing video games in the United States (Gottfried and Sidoti, 2024). Surprisingly and contrary to gender- and age-related stereotypes (Kowert et al., 2014), 41% of gamers are female and the average age of a video gamer is 36 (Entertainment Software Association, 2024). The increasing participation of adults in video gaming has been attributed to two reasons, the first being the fact that the first serious video gamers (i.e., those that were children when it first became popular) continue to play video games throughout their lives (Allaire et al., 2013; Brown, 2016, 2017). Secondly, video games are increasingly being designed to meet the specific social, existential, or health related needs of their players, such as games that alleviate loneliness or those that are used for physical therapy (Marston et al., 2016).

With the rise in video gaming’s popularity came a consequent rise in the need for social interactions, ranging from physical gatherings focused on a specific game to in-game communication and community management platforms like Discord and Twitch (Clark et al., 2015; Molyneux et al., 2015; Siitonen, 2015). Discord is a multi-channel (i.e., voice, video, text) communication platform which has become a central hub for players of specific games, within which they can discuss gameplay, find partners, and create organized communities. Twitch, the most popular video game streaming platform on the internet, allows gamers to become content creators and broadcast their games while directly interacting with their fans (Brewer et al., 2020; Brown and Moberly, 2020; Churchill and Xu, 2016).

The transformation of communication mechanisms within gaming has significantly enhanced their social dimensions. Online multiplayer games foster collaboration, competition, and the exchange of goods (Frommel et al., 2020; Jung, 2020). Social networking features, such as sharing achievements or in-game events on social media, further enhance gaming’s social dimension (Mäyrä, 2015). Gaming companies actively incorporate social interactions, such as in-game chat features, and develop in-game communities (often called guilds) where players can socialize and collaborate (Crenshaw and Nardi, 2015; Hou, 2011; Konert et al., 2014; Kuipers et al., 2018; Paavilainen et al., 2016; Richter, 2014). Thus, gaming has transcended mere entertainment, emerging as a cultural phenomenon and a vibrant hub for social interaction (Sublette and Mullan, 2012).

### 1.2 Gaming communities and their inherent paradox

Gaming communities are social networks formed around a common interest in gaming activities or specific games, providing spaces where players collaborate, form guilds, and engage in social interactions that frequently transcend the confines of the game (Mäyrä, 2015). Often described as “communities of interest” or “elective communities,” their members are driven by a shared enthusiasm for gaming (Brint, 2001; Mäyrä, 2015; Tushya et al., 2023). The structure and size of gaming communities vary significantly, catering to different needs such as aiding in-game activities, exchanging information, or socializing (Mäyrä, 2015; Williams, 2006). Nevertheless, gaming communities share several characteristics: they revolve around games, prioritize effective gameplay, offer anonymity (or at the very least pseudonymity, as online user information can still be used to “dox” players; Kowert, 2020), and facilitate forming social connections (Mäyrä, 2015; Saldanha et al., 2023). Furthermore, the online setting enhances their role as social catalysts by enabling multilingual and culturally diverse players to socialize and develop skills over time (Thorne et al., 2009).

However, these advances in social opportunities are not without their costs. One the one hand, gaming, like many other domains in the digital age (Araújo et al., 2017; Voida et al., 2009; Westin et al., 2015), is a nearly universally accessible activity (nearly only because individuals still need the money and consoles to play most games), open to all regardless of race, gender, or ethnic identity (Cimolino et al., 2021; Garcia and de Almeida Neris, 2020). On the other hand, it seems that the same social dynamics of exclusivity, out-group bias, and selectiveness, all exist and are sometimes amplified within gaming communities (Gray and Leonard, 2018; Molyneux et al., 2015; Siitonen, 2015). Thus, this review aims to examine the ways in which gaming communities are exclusionary and will provide guidelines to promote greater inclusivity.

### 1.3 The importance of inclusion in gaming communities

Investigating and fostering inclusivity in gaming communities is crucial for several reasons. First, gaming is prevalent among teenagers (Gottfried and Sidoti, 2024). As such, gaming communities have the potential to instill values and influence the social development of young people. Fostering cultural values of inclusivity may foster positive social norms and behaviors that extend beyond the gaming context (Cimolino et al., 2021; Garcia and de Almeida Neris, 2020). Second, gaming can significantly aid in the integration of marginalized individuals. For example, a recent study demonstrated how participation in a video game community offered immigrant Karenni children opportunities for language socialization and social connection (Duran, 2017).

Third, gaming communities influence innovations and advancements in the digital domain. According to the Diffusion of Innovations theory (Rogers, 1995; Rogers et al., 2014), innovators and early adopters of technology are key to the success of technological and social advancements. Gaming communities are unique in that they often foster and attract participants who enact innovator and early-adopter roles in digital diffusion processes (Gray, 2012a; Lin et al., 2007), and are thus highly impactful in setting social and technological trends across the digital realm (Attaran et al., 2019; Burger-Helmchen and Cohendet, 2011; Herodotou, 2010; Hussain and Griffiths, 2009; Jansz and Martens, 2005). A clear example comes from game modding communities, in which players create new maps, mechanics, and even total conversions; several commercially successful titles and genres have roots in such mod ecosystems (e.g., Counter-Strike from a Half-Life mod, and games inspired by the Defense of the Ancients mod for Warcraft III). Research further shows that modders gain recognition and influence through visible contributions, role specialization, and community collaboration, which helps explain how some participants come to occupy “innovator” roles, while others function as “early adopters” by rapidly testing, showcasing, and diffusing new content within the community and beyond (Thiel and Lyle, 2019). Fourth, gaming communities are often incorrectly overlooked targets of scholarly investigation, as they offer rich and accessible datasets for exploring social trends, group dynamics, and technological influence (Mäyrä, 2015; Rodrigues and Mustaro, 2007). As an example of this potential, the current review relies on data and analyses sourced from the gaming community in order to better understand the social dynamics of exclusion and inclusion (Frommel et al., 2020; Jung, 2020).

Finally, the Gamergate scandal and its consequences emphasize the importance of monitoring social trends in the digital space, and taking proactive measures towards achieving greater inclusiveness in gaming culture. Gamergate,^1^ initially a controversy over journalistic ethics in gaming, evolved to become a series of high-profile attacks on the advocates of diversity and inclusion within the gaming community. These attacks transformed into a movement which organized harassment campaigns primarily against gender, sexual, and ethnic minorities, leading to a deep division within the gaming community. This event exposed the downsides of the anonymity and ease of collaboration that the digital world provides, emphasizing the need for stronger community management and structural safeguards within this world. Nevertheless, Gamergate has also brought upon a surge of renewed interest and effort toward inclusivity (Braithwaite, 2016; Gray and Leonard, 2018; Martínez, 2020). This review continues this surge and examines both the barriers and potential enhancers of inclusivity, with a specific focus on the role played by gaming communities as catalysts for positive social change (Araújo et al., 2017; Voida et al., 2009).

### 1.4 The current study

This paper offers a detailed understanding of the current state of diversity and inclusion within gaming communities. Based on the insights derived from the literature, this review will propose strategies for solutions and identify areas requiring further study to advance inclusivity within gaming communities. Furthermore, the paper will delineate areas where additional studies are necessary to foster an inclusive culture within gaming. For detailed definitions and conceptualizations of key terms such as “gaming,” “community,” “culture,” “gaming communities culture,” and “social exclusion,” please refer to Supplementary materials. These definitions provide the necessary context for understanding the discussions in this paper and are available to enhance the clarity and precision of the terms used throughout the text.

## 2 Review methodology

Our literature review methodology echoes Kraus et al.’s (2020) systematic literature review guidelines. Compared to non-transparent, biased, and subjective reviews, systematic literature reviews are methodologically transparent, reproducible, and objective. The most important steps are: (1) formulating research questions; (2) developing a review protocol; (3) identifying the studies according to inclusion/exclusion criteria; and (4) extracting and synthesizing the data based on topics, not authors (Kraus et al., 2020).

### 2.1 Research questions

This review attempts to answer the following questions:

- What practices within gaming communities contribute to exclusion, and how do these exclusionary practices manifest across various gaming spaces?
- What initiatives or strategies have been implemented to promote inclusion within gaming communities, and what evidence exists regarding their effectiveness?
- What guidelines can be developed for future strategies and initiatives that enhance inclusivity in gaming communities?

### 2.2 Search strategy

The electronic search incorporated the keywords “gaming” or “gamer*” and “community” or “communities” with concepts that relate to inclusion and exclusion: “ethnic*” OR “gender*” OR “Race*” OR “age*” OR “diversity*” OR “inclus*” OR “exclus*” OR “representat*” OR “minorit*” OR “marginaliz*.”

We searched ProQuest, EBSCO Academic Search Complete, and Web of Science Databases (183, 349, and 298 articles, respectively) for published empirical papers. We added an additional paper found in Google Scholar during our general search. It is noteworthy that many interesting works in this field have been published in books, past reviews, and other forms that are outside the scope of this specific review.

**Figure 1:**
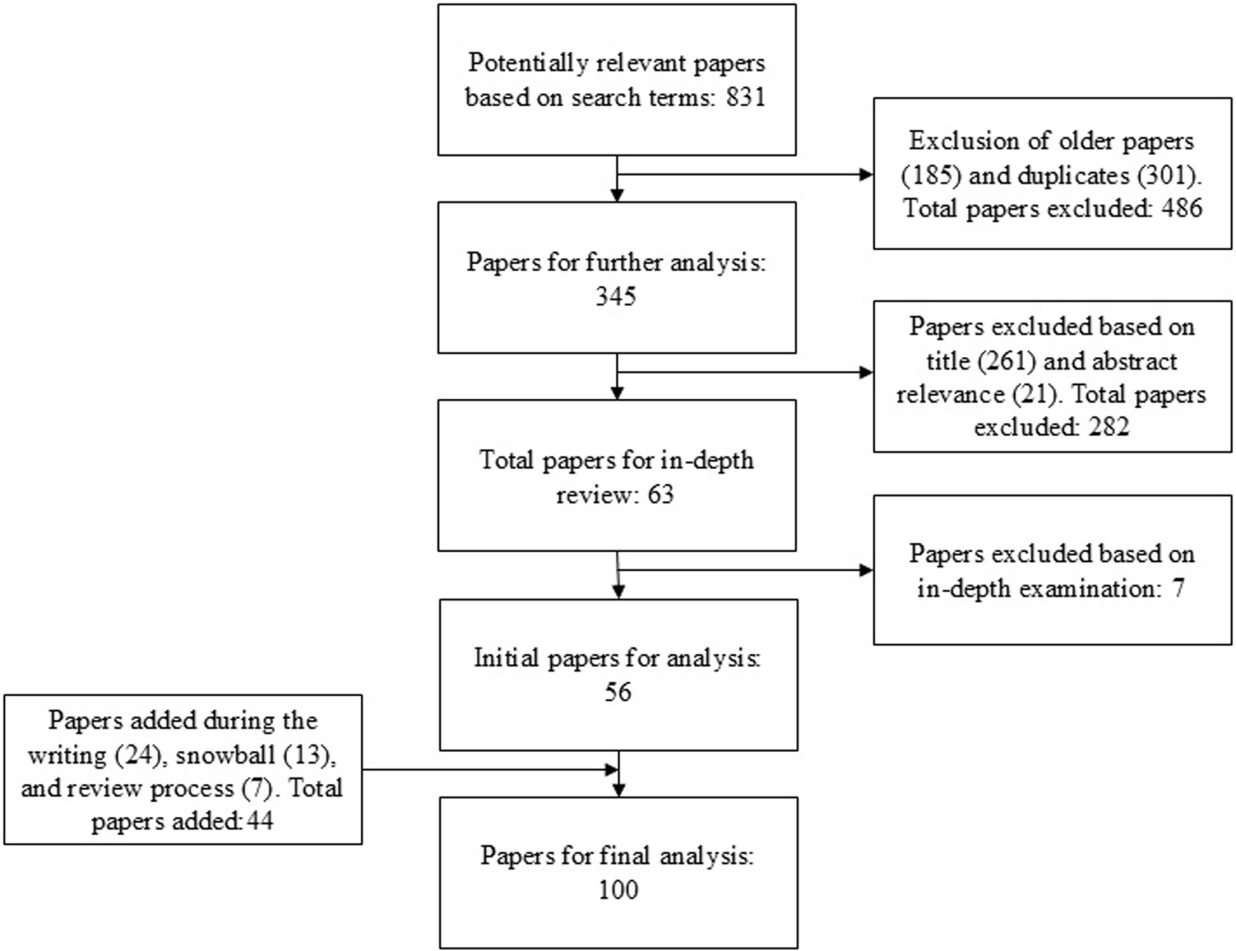
Literature review electronic search iterations.

Once we had our initial pool, we removed papers published prior to 2013 and duplicates (see Figure 1). The initial search was conducted at the beginning of 2024; therefore, we included papers published between 2013 and 2023 to ensure the review reflected the current evidence in a rapidly changing virtual ecosystem. Given the pace of technological change in digital environments and the evolving manifestations of social justice concepts such as inclusion and exclusion, we aimed to prioritize findings that are timely and relevant. We then excluded papers based on their titles and abstracts, and finally, on an in-depth examination.

To maintain relevance, we added additional relevant papers and new papers (from 2024 to the beginning of 2026) during the writing and review process. At this stage, two central papers from before 2014 that were removed due to their publication year were returned to the papers pool. Additionally, during the review process we conducted a snowball search to complement our original search method and avoid significant blind spots. The snowball included backward and forward snowballing using “CitationChaser” (Haddaway et al., 2022), seeking additional papers cited by central papers in the review and by those that cited the central papers. The papers were then added to create the final review pool (see Figure 1).

## 3 Results

This section aims to investigate the current state of inclusion and exclusion within online gaming communities by analyzing findings from scholarly articles published within the past decade. The subsequent section builds upon these insights, proposing concrete steps to foster inclusion within the gaming community.

### 3.1 Challenges and barriers to inclusion and diversity in gaming communities

#### 3.1.1 Social exclusion of women

One of the most pervasive challenges to achieving inclusivity within gaming communities is the persistence of gender-based stereotypes and discrimination towards women. These issues manifest themselves in various forms, contributing to an environment that is often hostile and unwelcoming for women gamers.

##### 3.1.1.1 Stereotypical representations of women gamers

Women gamers are often represented in a negative light, lacking key, socially desirable traits such as sociability, competence (Bustos-Ortega et al., 2023; Kelly et al., 2023; Morgenroth et al., 2020; Yao et al., 2023), and even being perceived as more “masculine” than non-gamer women (Yao et al., 2023). These stereotypes not only contribute to harmful gender biases, but also lead to the marginalization and exclusion of women within the gaming ecosystem. Interestingly, one study found that women gamers who used a male in-game avatar were perceived as being more competent (Kaye et al., 2017), underscoring a broader issue of gender bias in gaming.

##### 3.1.1.2 Oversexualization and objectification of women characters

Women are underrepresented as characters in video games, although players show little preference for the sex of the games’ protagonist, favoring advantageous or likable traits (la De Torre-Sierra and Guichot-Reina, 2025; Hanus and Dickinson, 2019; Jones et al., 2025). When women protagonist characters do appear, they are over-sexualized and objectified, with character designs including exaggerated physical features, revealing cosmetic attire, and suggestive poses all catered to a male audience (Boudreau, 2022; Lavigne, 2015; Morgan et al., 2020). Designs such as these reduce women characters to mere objects of desire, thus undermining their agency, personhood, and importance as characters in the video game world (la De Torre-Sierra and Guichot-Reina, 2025; Perry, 2022). While doing so may seem beneficial to the male players, it contributes to the greater normalization of sexual objectification and enhances the stereotype that the world of gaming is predominantly male.

##### 3.1.1.3 Prevalence of sexism and harassment

The stereotypical representations and objectification interact with sexism and harassment faced by women gamers, ranging from overt verbal abuse and discrimination to more covert and insidious practices that reinforce stereotypes and marginalization (Robinson, 2023; Saldanha et al., 2024; Silva et al., 2023; Sobieraj, 2018). Aggressors in online gaming forums employ intimidation, shaming, and discreditation tactics to hurt women and undermine their contributions (Jubran et al., 2025; Sobieraj, 2018). One common strategy is the weaponization of femininity by referencing women’s bodies to reassert gender differences and alleged male advantages within gaming spaces (Sobieraj, 2018). In one study of the forums and in-game communications within the highly popular online competitive game League of Legends, researchers found that women are often presumed to possess lesser skills, competence, and lower interest in gaming, thus actively discouraging their participation and advancement (Paaßen et al., 2017).

Research has extended beyond examining surface-level behaviors to investigate the underlying psychological mechanisms that drive cyber-aggression toward women in gaming environments (Fox and Tang, 2014; Jagayat and Choma, 2021). Social dominance orientation predicted video game sexism (Fox and Tang, 2014; Tang and Fox, 2016). Interestingly, sexism was not a predictor of perceiving women gamers as less competent or warm (Kelly et al., 2023). However, both social dominance orientation and right-wing authoritarianism were associated with cyber-aggression toward women; these effects were partially mediated by various types of threat (Jagayat and Choma, 2021). Perceived norms were also associated with making prejudiced remarks in online gaming. Importantly, the more normative they found confronting prejudiced remarks, the more they tended to report doing so (Cary et al., 2020). Identity fusion with the gamer identity, especially among insecurely attached, lonely individuals, may also lead to support for extremist behaviors associated with rejection of outgroups and minorities (Kowert et al., 2022).

In a survey conducted among women gamers, about 56% reported experiencing sexual harassment during online gaming (Zhou and Peterson, 2025). A comprehensive survey of World of Warcraft players exposed more alarming results with nearly two-thirds (63.6%) of women reporting experiences of sexism during gameplay (Brehm, 2013). Even more troubling, the majority (72.5%) of male participants failed to recognize sexism as a serious concern within the gaming environment. Some progress in this field was made as gaming culture became aware of feminist game scholars (Chess and Shaw, 2015). Despite this development, the disparity still exists, and highlights a critical challenge: while extensive research has shown that women gamers frequently experience sexism while playing, male gamers seem to be unaware of this issue and its severity to a large degree (Wells et al., 2025). This creates a significant barrier to developing inclusive gaming environments that encourage and support women’s participation.

##### 3.1.1.4 Impact on women gamers’ participation and experiences

The widespread sexism and toxic atmospheres significantly affect how women gamers participate in and experience gaming, influencing their behavior, sense of community connection, overall gaming satisfaction, and even mental health outcomes like increase in anxiety and loneliness (Di Leo et al., 2025; McLean and Griffiths, 2019; Trudgett-Klose and McLinton, 2024; Vergel et al., 2024). Some women gamers avoid communication (like in-game chat) with other gamers, thus avoiding inflammatory communication, but also missing out on joint strategizing and community (Fox and Tang, 2017; Ratan et al., 2015). Some research found no association between stereotype threat and gaming performance or game-related self-efficacy (Pennington et al., 2018). Yet other papers found that women face the exhausting burden of continuously demonstrating their competence, defending their expertise, and challenging prevailing stereotypes, pressures that can discourage them from pursuing gaming interests or competing at higher levels (Saldanha et al., 2024). As their contributions are overlooked, dismissed, or received with shock, women’s feelings of exclusion intensify. This cycle of marginalization can foster alienation and undermine self-confidence, ultimately driving women away from certain gaming communities (Braithwaite, 2014; Brooker, 2026; Saldanha et al., 2024).

Women who remain in these gaming environments develop various coping mechanisms. Research on the Facebook game YoWorld documented how women players experienced bullying, identity theft, and public humiliation (Bergstrom, 2021; McLean and Griffiths, 2019). In response, some actively hide their identity, or withdraw from broader community participation, and seek exclusive, protected spaces where they can game safely and receive support from other women (Bergstrom, 2021; Fox and Tang, 2017; McLean and Griffiths, 2019; Zhang et al., 2023). The effects of sexism and harassment often emerge in more subtle ways as well (Giolla Easpaig and Humphrey, 2017). For example, research on women’s experiences in online video games shows that players often adopt strategic coping practices such as playing only when their partner is playing with them, concealing gender identity, avoiding voice or text communication, selectively disclosing personal information, and carefully managing how they present themselves to other players in order to reduce harassment exposure (Cote, 2017; Ratan et al., 2015).

#### 3.1.2 Racial and ethnic underrepresentation and stereotyping

##### 3.1.2.1 Lack of diversity in game characters and narratives

Racial and ethnic minorities face significant barriers to inclusion and representation within gaming communities. Studies indicate that non-whites are underrepresented as characters in video games (Dietrich, 2023; Jones et al., 2025), and the non-white characters that do exist are often depicted through stereotypical tropes or problematic representations. Many games fail to incorporate characters and storylines that reflect the experiences and perspectives of racial and ethnic minorities, depriving players of color from seeing themselves represented and acknowledged within these immersive environments (Devereaux, 2023; Richard and Gray, 2018; Smith and Thakore, 2023). This lack of representation is exceedingly problematic, as non-white male participants prefer to select characters like themselves (Kukshinov and Shaw, 2022). When racial and ethnic minorities are represented within games, their depictions often rely on harmful stereotypes and problematic tropes (Richard and Gray, 2018; Smith and Thakore, 2023). For example, an analysis of popular video games found that Black male characters frequently embodied racist caricatures like criminals, athletes, or minstrel depictions. In contrast, Black women were portrayed through “Jezebel” or “Sapphire” stereotypes (Smith and Thakore, 2023). Such one-dimensional and stereotypical representations not only reinforce prejudiced viewpoints but also fail to capture the nuanced and diverse experiences of racial and ethnic minorities. Some ethnic minority gamers choose identity concealment among other in-game coping strategies (Passmore and Mandryk, 2020), this strategy is even more pronounced among players with compound marginalization having more than one marginalized identity (Phillips, 2025). However, gamers of color who wish to express their ethnic identity spend more money on alternative “skins,” cosmetic changes to the default player avatar, because most default, free avatars are white (Reza et al., 2022).

##### 3.1.2.2 Discrimination, racism, and prejudice in gaming communities

The lack of authentic representation along with the prevalence of stereotypical portrayals directly contributes to experiences of racism, discrimination, and marginalization for gamers of color within gaming communities. Studies have documented extensive patterns of racial harassment, hate speech, and discriminatory treatment targeting these players (Gray, 2012b; Ghosh, 2021; Kowert et al., 2022; Richard and Gray, 2018). One study found that dark-skinned avatars were evaluated more positively than light-skinned ones (Vang and Fox, 2014). Despite this surprising finding, it seems that players perceive in-game prejudiced behavior as more acceptable and normative than real-life prejudice, which may contribute to the prevalence of these phenomena (Cary et al., 2020; Cary and Chasteen, 2022). The victims of racist hate speech in the form of trash talk may simply attempt to desensitize themselves to this type of speech, as they face external views that do not see these experiences as “real” racism (Ortiz, 2019). The combination of underrepresentation, stereotypical depictions, and experiences of discrimination creates a sense of profound alienation for gamers of color, who frequently report feeling unwelcome and displaced within gaming spaces (Passmore and Mandryk, 2020; Richard and Gray, 2018). The damages of racism in online gaming extend beyond the virtual environment, as minority players’ exposure to online racism is linked to higher psychological distress (TaeHyuk Keum and Hearns, 2022). These barriers prevent positive community engagement and participation, reinforcing the perception that gaming environments are designed exclusively for white audiences.

#### 3.1.3 LGBTQ+ marginalization and exclusion

##### 3.1.3.1 Lack of representation, heteronormativity, and homophobia in gaming culture

While queer game characters and plot lines do exist (Shaw and Friesem, 2016), the lack of representation and inclusion within many game narratives, character designs, and customization options is still a significant barrier faced by LGBTQ+ gamers (Anglada-Pujol, 2022; Gray, 2018; Krobová et al., 2015; Shelton et al., 2023). Moreover, the culture of many gaming communities is deeply rooted in heteronormative assumptions and gender binaries that effectively erase or exclude LGBTQ+ identities and experiences (Crowe and Watts, 2014; Jubran et al., 2025; Krobová et al., 2015; Robinson, 2023; Shaw, 2017). Compounding this issue is the prevalence and normalization of homophobic language and anti-LGBTQ+ attitudes within gaming communities (Cote, 2018; Crowe and Watts, 2014; Meric, 2026), and the fact that LGBTQ+ gamers receive more negative feedback and encounter more hate speech than non-LGBTQ+ gamers (Gillin and Signorella, 2024). In one telling yet commonplace example, a YouTube video shows the use of homophobic slurs such as “faggot” as a response to negative gameplay experiences (Two Lonely Penguins, 2025). A basic search of YouTube for homophobic slurs in video games produces countless similar examples.

The combination of marginalization through invisibility, lack of representation, and direct discrimination creates a profoundly unwelcoming environment for LGBTQ+ individuals within gaming communities (Brenner-Levoy, 2023). Many of these gamers are forced to choose between concealing their sexual orientation and/or identity to avoid discrimination, and being open about who they are and risk harassment (Gray, 2018; Shaw, 2017). This tension has driven many LGBTQ+ gamers to self-censor, disengage from the gaming community, and even quite certain games entirely (Perry, 2022). This dynamic perpetuates a cycle of exclusion and marginalization, hindering the potential for gaming to be a truly inclusive and welcoming space for individuals of all sexual orientations and gender identities. Some studies examined the positive effects of supportive gaming environments that allow LGBTQ+ self-expression (Hasei et al., 2025). The striking contrast between the current hostile environment and the potential of gaming as a supportive platform for LGBTQ+ youth stresses the need to address the diverse challenges faced by LGBTQ+ gamers (Shaw and Friesem, 2016).

### 3.2 Strategies and initiatives for promoting inclusion and diversity

Fostering inclusive and diverse gaming communities requires a multifaceted approach involving various strategies and initiatives across different levels, from game design and development to community management, grassroots activism, and industry partnerships.

#### 3.2.1 Inclusive game design and development

##### 3.2.1.1 Emergence of non-traditional narratives and diverse perspectives

One key aspect of resistance and resilience among marginalized gamers is creating and circulating counter-stereotype narratives (Naidoo et al., 2020). Harvey (2014) provides a relevant example of the shift in gaming culture reflected in Twine. Twine is an open-source software tool designed to craft interactive narratives and hypertext fiction. It empowers users to construct branching stories, often used in games, where players navigate the narrative’s flow by making choices that influence the plot’s direction. The study examined how Twine game makers provide queer alternatives to traditional digital game culture by subverting assumed norms in game design and distribution. Another example of a game that challenges traditional narratives and character designs is Animal Crossing: New Horizons (Shelton et al., 2023). This game allows players to customize their character’s gender expression and presentation, offering them opportunities to engage in queer and trans-affirming worldmaking through its in-game mechanics and player-created online communities.

##### 3.2.1.2 Avatar diversity and flexibility

While characters are fixed entities within games, avatars function as digital extensions of players, providing opportunities for personalization and identity expression. Research demonstrates that game developers can create more inclusive gaming experiences by emphasizing diverse character options and extensive customization features (Lavigne, 2015; Morgan et al., 2020; Perry, 2022; Shelton et al., 2023). Avatars enable players to express their identities, cultural backgrounds, and personal preferences, fostering a stronger sense of community (Teng, 2017). Allowing greater flexibility and diversity in avatar creation not only increases player engagement and satisfaction, but also communicates a clear commitment to inclusivity within virtual environments (Birk et al., 2016; Koulouris et al., 2020).

##### 3.2.1.3 Diverse development teams and voices

Background and expertise diversity in teams can enhance the creative process, leading to more novel ideas, more unique narratives, and the exploration of previously overlooked or underrepresented themes and perspectives (Richard and Gray, 2018; Smith and Thakore, 2023). For instance, a study found that games developed by diverse teams consistently feature more sophisticated and genuine representations of marginalized communities, resulting in richer and more engaging gaming experiences (Boudreau, 2022). However, it seems that some diversity, equity, and inclusion initiatives in the field tend to be more performative than formative and have limited impact (Darvin et al., 2025).

#### 3.2.2 Community management and moderation

##### 3.2.2.1 Creation of online communities among marginalized players

Findings indicate that marginalized gamers have actively cultivated alternative gaming spaces and communities that foster a sense of belonging, support, and empowerment (AlAfnan, 2025; Gray, 2018). Games with diverse characters and narratives, such as “Animal Crossing: New Horizons” specifically tend to foster the creation of online communities among marginalized players (Blanco-Fernández and Moreno, 2023; Gandolfi and Gandolfi, 2021; Shelton et al., 2023), which offer a sense of belonging and acceptance and enable forms of creative self-expression and counter-gaming practices. Bergstrom (2021) underscored such communities’ positive role during the COVID-19 pandemic as a valuable socializing tool for teenagers and young adults to navigate the challenges of a turbulent time and foster social connections and inclusion at a time when physical distancing measures limited in-person interactions.

By incorporating inclusive content, games initiate a positive cycle that enriches representation and fosters safe spaces for marginalized groups. This, in turn, attracts more diverse players, leading to a more inclusive gaming culture. The cycle perpetuates as increased representation encourages the development of more inclusive content, which further expands safe spaces.

##### 3.2.2.2 Management and moderation practices

Effective community management and moderation practices play a vital role in shaping the culture and norms within gaming communities. One key strategy involves establishing and enforcing clear codes of conduct that outline guidelines and consequences for harassment, discrimination, and other unacceptable behavior (Cote, 2018; Robinson, 2023; Zorlu and Özkan, 2020). These codes signal a commitment to creating safe, welcoming environments for all participants. Another critical aspect is the active identification and mitigation of toxic behaviors, harassment, and discrimination within gaming spaces (Srikasetsarakul and Vungthong, 2019). Effective moderation requires robust reporting systems that enable players to flag violations and abuse (Bergstrom, 2021; Graham, 2019; Robinson, 2023), coupled with consistent enforcement mechanisms ranging from warnings to permanent account suspensions (Anglada-Pujol, 2022). In addition, proactive approaches are equally important for preventing and mitigating toxicity within gaming communities, such as providing education and awareness campaigns, and fostering a culture of respect and inclusivity (Cook et al., 2024; Cote, 2018; Jagayat and Choma, 2021; Robinson, 2023; Sanghvi et al., 2024; Sobieraj, 2018). For example, moderators can promote positive community values, such as respect, empathy, and allyship, through regular messaging and community events.

Ultimately, effective moderation fundamentally transforms gaming culture through consistent rule enforcement, proactive intervention against toxic behavior, and active promotion of inclusive values, creating environments where all players experience safety, respect, and belonging (Bergstrom, 2021; Robinson, 2023). Moderators can champion marginalized community members by elevating their perspectives, addressing their concerns, and tackling discrimination or exclusion directly. This advocacy role extends to collaborative efforts with players, community organizations, and industry stakeholders to develop comprehensive strategies that advance inclusivity and diversity throughout gaming spaces.

##### 3.2.2.3 Industry initiatives and partnerships

Many gaming companies have recognized diversity’s importance and implemented initiatives and partnerships to address these issues. One key strategy has been implementing inclusive hiring and retention practices (Richard and Gray, 2018; Zorlu and Özkan, 2020) to ensure a wide range of perspectives and experiences. Additionally, some companies have established dedicated diversity and inclusion departments and initiatives (Gray and Leonard, 2018; Smith and Thakore, 2023) to help create a more welcoming and inclusive environment for all employees and players. Finally, gaming companies have formed strategic partnerships with diverse organizations and advocacy groups to elevate marginalized voices, incorporate valuable community insights, and ensure authentic representation of underrepresented experiences within the industry (Boudreau, 2022; Richard and Gray, 2018; Zorlu and Özkan, 2020).

##### 3.2.2.4 Social justice advocacy

When prejudice is seen as normative, people accept it; when confronting it is seen as normative, people tend to confront it more (Cary et al., 2020). Studies reveal the growing influence of grassroots initiatives and player-driven communities that establish safe, inclusive environments for marginalized gamers (Anglada-Pujol, 2022; Gray, 2018; Shelton et al., 2023). For instance, LGBTQ+ players have created supportive online spaces like the “Gaymers” subreddit, where members share experiences, offer mutual support, and advocate for improved representation while organizing collective action against exclusionary practices (Shaw, 2017). Another study discussed the work of “I Need Diverse Games,” an organization that provides resources, mentorship, and support for marginalized creators (Boudreau, 2022). The study suggests that by amplifying underrepresented voices, such organizations can pressure the gaming industry to address these issues. Player activism extends beyond community building to direct action, as demonstrated by the #BoycottBlizzard movement, where participants employed innovative strategies including in-game protests and coordinated online campaigns to challenge corporate authority and demonstrate solidarity with political causes (Chamberlain, 2022). Another study examined the role of gaming podcasts as a form of grassroots media that can challenge dominant narratives (Robinson, 2023). The study argues that podcasts created by and for gamers from underrepresented groups can offer counter discourses that critique exclusionary practices, celebrate diverse gaming experiences, and push for greater inclusion and representation. Finally, serious games – or games designed with additional educational or social justice purposes other than entertainment – have the potential to lower interpersonal and intergroup prejudice and discriminatory attitudes (Olson and Harrell, 2020).

#### 3.2.3 Decoding the power and potential of algorithms

Algorithms are crucial in shaping virtual environments and influencing user interactions (Weber et al., 2014), including those related to inclusion and diversity within gaming communities. One study examined the impact of behind-the-scene algorithmic assortative matching (López Vargas et al., 2022), i.e., matching users with others who are similar to them in skill level, interests, etc. The findings revealed that while such algorithms aim to optimize gameplay experiences, they can inadvertently create barriers between novices and experts, reinforcing homogeneous community clusters. This pattern raises concerns about whether matching algorithms may similarly limit cross-demographic interactions, potentially segregating players by gender, ethnicity, or other background characteristics. Conversely, algorithms designed for content moderation show promise for promoting inclusivity through automated detection of hate speech, harassment, and discriminatory language within gaming environments (Sanghvi et al., 2024).

Algorithms can also serve as a means to monitor the broader environment within gaming communities. Studies demonstrate the potential of algorithms to analyze and track changes in the overall culture and social dynamics. For example, algorithmic curation of League of Legends gameplay showed that women and people of color were largely absent (Woodhouse, 2022). Additionally, Kim et al. (2018) highlighted how Twitter’s feed and algorithms can both amplify and constrain marginalized voices and potentially provide valuable insights into the impact of initiatives promoting inclusion and diversity.

## 4 Overview of results

The literature review has revealed several significant challenges and barriers to inclusivity within gaming communities (see Table 1). Prominent among these are the persistent stereotypical representations and marginalization of women, with prevalent sexism, harassment, and objectification contributing to hostile environments that discourage women participation (Bergstrom, 2021; Brehm, 2013; Hayday and Collison, 2020; Michalik, 2015; Perry, 2022; Robinson, 2023; Saldanha et al., 2024; Sobieraj, 2018; Yao et al., 2023). Similarly, racial and ethnic minorities face underrepresentation, stereotypical depictions, and experiences of discrimination within gaming spaces (Devereaux, 2023; Gray, 2012b; Richard and Gray, 2018; Smith and Thakore, 2023). The LGBTQ+ community also encounters marginalization through invisibility, lack of representation, and direct harassment based on sexual orientation and gender identity (Anglada-Pujol, 2022; Crowe and Watts, 2014; Gray, 2018; Perry, 2022; Robinson, 2023; Shaw, 2017; Shelton et al., 2023). The review also underscores a general need for more awareness regarding the experiences of marginalized gamers, insufficient transparency and accountability in the algorithms that influence the gaming realm, and a notable dearth of regulation. At the same time, the review also offers hope by highlighting existing initiatives in promoting inclusivity within gaming communities.

**Table 1:** Findings Summary.

Issue	Key findings	Papers
Social exclusion of women and girls	- Gender-based stereotypes - Objectification of female characters—Sexism and harassment toward female gamers—Hostile environment discouraging participation	AlAfnan (2025), Ballard and Welch (2017), Bergstrom (2021), Braithwaite (2014), Brehm (2013), Brooker (2026), Bustos-Ortega et al. (2023), Cary and Chasteen (2022), Cary et al. (2020), Chess and Shaw (2015), Cote (2017), Cote (2018), Crowe and Watts (2014), la De Torre-Sierra and Guichot-Reina (2025), Di Leo et al. (2025), Ekiciler et al. (2022), Fox and Tang (2014), Fox and Tang (2017), Gandolfi et al. (2023), Ghosh (2021), Graham (2019), Gray (2018), Hanus and Dickinson (2019), Hayday and Collison (2020), Jagayat and Choma (2021), Jones et al. (2025), Jubran et al. (2025), Kaye et al. (2017), Kelly et al. (2023), Kim et al. (2018), Lavigne (2015), Martínez (2020), McKinnon-Crowley (2020), McLean and Griffiths (2019), Meric (2026), Michalik (2015), Morgan et al. (2020), Naidoo et al. (2020), Giolla Easpaig and Humphrey (2017), Paaßen et al. (2017), Passmore and Mandryk (2020), Pauser and Wagner (2020), Pennington et al. (2018), Perry (2022), Phillips (2025), Ratan et al. (2015), Richard and Gray (2018), Robinson (2023), Saldanha et al. (2024), Shelton et al. (2023), Silva et al. (2023), Sobieraj (2018), Tang and Fox (2016), Tang et al. (2020), Trudgett-Klose and McLinton (2024), Vergel et al. (2024), Weber et al. (2014), Wells et al. (2025), Wong and Ratan (2024), Woodhouse (2022), Yao et al. (2023), Zhang et al. (2023), Zhou and Peterson (2025), Zorlu and Özkan (2020)
Racial and ethnic underrepresentation and stereotyping	- Lack of diversity in game characters and narratives - Experiences of racism and discrimination faced by gamers of color	AlAfnan (2025), Bergstrom (2021), Cary and Chasteen (2022), Cary et al. (2020), Devereaux (2023), Dietrich (2023), Duran (2017), Ghosh (2021), Graham (2019), Gray (2012b), Gray (2018), Jones et al. (2025), Jubran et al. (2025), Kim et al. (2018), Meric (2026), Olson and Harrell (2020), Ortiz (2019), Passmore and Mandryk (2020), Pauser and Wagner (2020), Phillips (2025), Reza et al. (2022), Richard and Gray (2018), Smith and Thakore (2023), TaeHyuk Keum and Hearns (2022), Vang and Fox (2014), Weber et al. (2014), Wells et al. (2025)
LGBTQ+ marginalization and exclusion	- Heteronormative assumptions and homophobia - Lack of representation and inclusion - Overt discrimination against LGBTQ+ gamers	Anglada-Pujol (2022), Ballard and Welch (2017), Bergstrom (2021), Blanco-Fernández and Moreno (2023), Brenner-Levoy (2023), Brooker (2026), Cary and Chasteen (2022), Cary et al. (2020), Crowe and Watts (2014), Ghosh (2021), Gillin and Signorella (2024), Gray (2018), Harvey (2014), Hasei et al. (2025), Jubran et al. (2025), Krobová et al. (2015), Kukshinov and Shaw (2022), Meric (2026), Morgan et al. (2020), Paaßen et al. (2017), Passmore and Mandryk (2020), Phillips (2025), Shaw and Friesem (2016), Shaw (2017), Shelton et al. (2023), Weber et al. (2014), Wells et al. (2025)
Lack of awareness	- Lack of awareness and recognition among some gamers, particularly male players, regarding the prevalence of harassment and misrepresentation faced by marginalized groups	Cary and Chasteen (2022), Chamberlain (2022), Chess and Shaw (2015), Olson and Harrell (2020), Passmore et al. (2018), Srikasetsarakul and Vungthong (2019)
Generalized Outgroup Hostility	- Discriminatory behavior is perceived as more normative when it occurs online than face-to-face - The more prejudice is seen as normative, the more likely it is to occur	Ballard and Welch (2017), Brooker (2026), Cary and Chasteen (2022), Cary et al. (2020), Ghosh (2021), Jubran et al. (2025), Kowert et al. (2022), Passmore and Mandryk (2020), Wells et al. (2025)
Strategies and Initiatives for Promoting Inclusion and Diversity	- Inclusive game design - Community management and moderation - Industry initiatives and partnerships - Social justice advocacy - Algorithms and data-driven approaches	Anglada-Pujol (2022), Babichenko et al. (2023), Birk et al. (2016), Blanco-Fernández and Moreno (2023), Boudreau (2022), Braithwaite (2016), Chamberlain (2022), Cook et al. (2024), Darvin et al. (2025), Duran (2017), Fox and Tang (2017), Gandolfi and Gandolfi (2021), Graham (2019), Gray (2012b), Gray (2018), Harvey (2014), Hasei et al. (2025), Hayday and Collison (2020), Jagayat and Choma (2021), Kim et al. (2018), Koulouris et al. (2020), López Vargas et al. (2022), Martínez (2020), Olson and Harrell (2020), Ortiz (2019), Passmore and Mandryk (2020), Passmore et al. (2018), Pauser and Wagner (2020), Perry (2022), Sanghvi et al. (2024), Shaw (2017), Shelton et al. (2023), Smith and Thakore (2023), Teng (2017), Weber et al. (2014), Woodhouse (2022), Zorlu and Özkan (2020)

## 5 Solutions and recommendations

The following sections elaborate on the existing initiatives while suggesting new strategies to foster inclusion and diversity within gaming communities. Recognizing that the gaming ecosystem comprises several key players - policymakers, gaming companies, leading community members etc. - these strategies address the role of each stakeholder. In addition, we explore how these “players” can collaborate to create a more inclusive gaming environment. Finally, we point to the importance of academic research, as it can be crucial in evaluating the effectiveness of existing strategies, identifying improvement areas, and proposing evidence-based solutions. Moreover, research can shed light on the complex dynamics and intersectionality of marginalization within gaming communities, providing a deeper understanding of the experiences and needs of diverse groups. By harnessing the collective efforts of all stakeholders in the gaming ecosystem while also leveraging the insights gleaned from rigorous academic inquiry, the gaming industry and its communities can work towards creating a more inclusive, equitable, and diverse gaming culture.

### 5.1 Creating safe spaces for vulnerable groups

The findings of the current review indicate that for many players from underrepresented groups the mainstream gaming community can be a daunting and even hostile place (Cote, 2018; Crowe and Watts, 2014; Gray, 2012b). The prevalence of toxicity, harassment, and discrimination in some gaming spaces can create significant barriers to entry and participation for these players (Gray, 2018; Passmore and Mandryk, 2020; Saldanha et al., 2024; Shaw, 2017). Safe spaces can help mitigate these challenges by providing an environment where players can explore, learn, and socialize without fear of judgment or harassment (Lucero, 2017; Vaccaro et al., 2011). These spaces can take the forms of gaming communities dedicated to specific marginalized groups, such as gamers of color (Gray, 2018), women gamers, LGBTQ+ gamers (Shelton et al., 2023; Tang et al., 2020), or gamers with chronic physical or mental conditions (Babichenko et al., 2023; Lucero, 2017). These communities offer not only a refuge from the toxicity often present in mainstream gaming spaces, but also provide a supportive and inclusive environment where players feel valued and respected (Blanco-Fernández and Moreno, 2023; Weigel and Rudnick, 2023). Research shows that a sense of belonging and social support reduces negative emotions such as isolation, anxiety, and depression (Fisher et al., 2015; Hagerty and Williams, 1999). Similarly, recent findings have documented that feelings acceptance and support can foster positive emotions and increase people’s positive mental health (Gray, 2018; Lucero, 2017; Shaw, 2017).

Safe spaces function as catalysts for integrating marginalized gamers into broader gaming communities by cultivating belonging, facilitating skill development and knowledge exchange, and mitigating negative emotional experiences that can inhibit participation (Vaccaro et al., 2011). Indeed, within these environments, novice players can receive mentorship from experienced community members, building both professional competence and emotional resilience while developing effective coping strategies(Gray, 2018). This peer-to-peer learning proves especially crucial for underrepresented players who often lack mentorship opportunities and may feel overwhelmed in mainstream gaming environments (Le Hénaff et al., 2015; Romero et al., 2012). The emotional benefits extend beyond confidence building, as chronic negative emotions—particularly shame—frequently manifest as anger and aggression in gaming contexts (Tangney et al., 1992). By reducing these adverse emotional experiences, safe spaces help regulate aggressive tendencies and enable participants to engage in more constructive, collaborative behaviors.

Safe spaces can be created and maintained by various actors within the gaming ecosystem. Game developers and publishers can establish dedicated servers, specialized forums, or curated environments within their platforms specifically designed to support underrepresented players. These companies can also amplify existing inclusive communities by partnering with or promoting welcoming Discord servers, subreddit communities, and fan-run forums that align with their games. Beyond corporate initiatives, grassroots efforts prove equally vital—community moderators, streamers, and dedicated players often spearhead the creation of these protective environments, building spaces where marginalized gamers can connect, compete, and collaborate without fear of harassment or discrimination.

### 5.2 Integration of diverse groups into mainstream gaming

While safe spaces play a valuable role, the ultimate goal of inclusivity is players’ integration into mainstream gaming communities. Accordingly, in the following sections, we outline solutions and recommendations while addressing the measures required from various stakeholders. Our discussion will explore how to foster collaboration between these stakeholders and transform their efforts into shared cooperation.

#### 5.2.1 Encouraging diversity through game design

Gaming companies, developers, and publishers hold a unique and powerful position in promoting diversity and inclusion. One approach is the inclusion of diverse characters, avatars, and narratives. Diverse representation in games can encompass a broad array of avatars and character customization options, such as the ability to select different skin tones, body shapes, and gender presentations (Morgan et al., 2020; Pauser and Wagner, 2020). Providing players with the tools to craft avatars that mirror their own identities and experiences can foster self-expression, a sense of belonging, and validation for marginalized players (Fordyce et al., 2018; Teng, 2017). Additionally, inclusive game design can extend to narrative and thematic elements. By weaving diverse stories, characters, and perspectives into game narratives, developers can encourage empathy, understanding, and respect for diversity within the gaming community.

Another promising approach that has received less attention in the scientific literature is the development of game mechanics that naturally bring together players from different backgrounds and skill levels (Mulak and Winiewski, 2021; Nardi and Harris, 2006; Vicencio-Moreira et al., 2015). Many of today’s games are cooperative and include tasks that require collaboration and teamwork (Farah et al., 2022; Pollok et al., 2021). For example, in the popular game Among Us, players work together to identify and vote out the “imposter” who is sabotaging the team’s efforts. This game mechanic naturally encourages communication and cooperation among players, regardless of their background or skill level. Similarly, Animal Crossing: New Horizons fosters community through its emphasis on mutual aid and shared experiences. Players visit each other’s virtual islands, exchange resources, and participate in collaborative events, with the game’s design actively rewarding cooperation and resource sharing. The game’s design emphasizes social cooperation, as players can help each other with tasks, share resources, and engage in friendly competition. These mechanics create a sense of community and shared experiences among players, regardless of their real-world identities or backgrounds (Adachi et al., 2016; Pech and Caspar, 2024).

Finally, an additional promising avenue that has yet to receive enough research attention is matchmaking systems (i.e., connecting players together for online play sessions) that prioritize diversity. Traditional matchmaking algorithms pair similarly skilled players in both competition and cooperation contexts (Boroń et al., 2020; Pramono et al., 2018). Nevertheless, research shows that matchmaking based on similar skill levels may not be as enjoyable for players as pairing them with lower skill-leveled counterparts (Kang et al., 2024), and researchers argue that matchmaking players based on additional characteristics unrelated to skill can possibly lead to greater enjoyability (Delalleau et al., 2012; Horton et al., 2016; Villar and León, 2022). These findings are troubling and promising. On one hand, if players prefer those similar to them, it implies less inclusion of marginalized groups. On the other hand, it might be possible to create more sophisticated and complex algorithms that find similarities between players from minority groups and those from mainstream groups. For example, gamers can be matched according to criteria such as their pet preferences (e.g., dogs vs. cats) or hobbies (e.g., rock climbing) instead of their belonging to potentially prejudiced social groups (e.g., straight vs. LGBTQ+).

#### 5.2.2 Encouraging diversity through industrial policies

Game publishers and platforms can also use their policies and incentive structures to encourage inclusive behavior and discourage toxicity (Lewis et al., 2016; Martínez, 2020). For example, players could earn special in-game items, currency, or upgrades for consistently demonstrating positive and inclusive behavior, such as reporting toxic behavior or participating in community-driven diversity initiatives. Additionally, platforms could feature player profiles who have significantly fostered inclusivity. Implementing such incentive systems can help create a culture of positivity regarding inclusion and diversity (Wang and Sun, 2011; Yin et al., 2022).

In addition, game publishers and platforms can also take measures to minimize and prevent harassment and toxic behaviors. One way can be by providing educational information. Educational policies are critical because, as findings show, many gamers are unaware of the prevalence of harmful practices and their effects on individuals from marginalized groups (Adinolf and Turkay, 2018; Komaç and Çağıltay, 2021). Furthermore, studies indicate that although most people view themselves as moral, many behave immorally while remaining unaware of their behavior, in a process called moral disengagement (Barkan et al., 2015; Moore, 2015). In such cases, knowledge regarding moral disengagement and the mere indication that exemplifies where one behaved immorally can profoundly alter one’s behavior for the better (Ayal et al., 2015; Bustamante and Chaux, 2014).

In addition, game publishers can establish clear consequences for toxic behavior, a more active and far-reaching strategy. That is, just as in the physical world, where alongside education for proper, legal, and ethical behavior, there are also legal and enforcement systems in place, so too in the digital reality of games, education may not suffice. Therefore, clear consequences, such as a communication channel mute, followed by a temporary ban and then a permanent ban, are needed for players who persistently engage in harassment or discrimination (Wijkstra et al., 2024). Measures such as these will not only punish violators and potentially discourage any further violations, but will also send a message to the community at large that such conduct will not be tolerated.

Beyond their ethical importance, the recommendations in Sections 5.1–5.3 also have a practical business rationale. Toxicity and harassment can undermine core aspects of the player experience that are directly tied to the success of multiplayer games, including enjoyment, relatedness, and social connection. For example, recent empirical work shows that perceived toxicity in gaming communities is associated with lower in-game social capital and lower satisfaction of relatedness needs, as well as higher loneliness; the authors explicitly note that these benefits of multiplayer play are often undermined by toxicity and that this is detrimental for developers when players leave their games (Frommel et al., 2023). This is especially important for retention. Prior research has argued that toxic behavior can worsen gameplay experiences and contribute to player churn, thereby threatening revenue and long-term game popularity (Kordyaka et al., 2020).

### 5.3 Regulatory measures for diversity and inclusion

Policymakers and regulatory bodies can play a pivotal role in ensuring that marginalized players’ interests are protected and that gaming companies’ social responsibilities are upheld. For example, existing laws against discrimination and harassment in the workplace and public spaces can be applied to online gaming environments (Phidd, 2018). This aims to empower players to report incidents of harassment and hold companies accountable for fostering a safe and inclusive environment (e.g., prohibiting stereotypical or offensive depictions). By establishing clear expectations and consequences for non-compliance, regulatory bodies can help drive meaningful change and accountability within the industry. However, enforcement presents significant challenges in the digital realm, including complex jurisdictional questions across international platforms, the persistent online anonymity that shields perpetrators, and inherent difficulties in documenting ephemeral online interactions as legal evidence (Phidd, 2018).

Soft regulation can also be instrumental (Blomqvist, 2022). This approach includes techniques such as publishing objective information at odds with companies’ or individuals’ desirable or ethical behavior. Currently, established rating systems like the Entertainment Software Rating Board (ESRB) in North America and the Pan European Game Information (PEGI) system warn players about potentially offensive or discriminatory elements. Specifically, the PEGI indicates whether a game contains depictions of ethnic, religious, nationalistic, or other stereotypes likely to encourage hate. The aim of such a label is to empower informed choices for players while avoiding direct censorship of game content. However, critics argue that these systems primarily focus on age-appropriateness and may not adequately capture the nuances of potentially harmful stereotypes or discriminatory portrayals within games (Felini, 2015; Lučić et al., 2022).

Some regulatory bodies propose requiring gaming companies to report on the prevalence of harassment and discrimination within their games. Recent studies highlight the application of soft regulation in promoting diversity within the gaming industry, illustrating its effectiveness (Király et al., 2018). Beyond the publication of undesirable information for the aim of regulatory shaming, policymakers can also publish inclusive and diversity-desirable information (Felini, 2015; Jerrett and Howell, 2022). For example, there are calls to implement badges or labels highlighting positive aspects of games. These badges or labels may indicate positive social messages, educational value, or prosocial gameplay mechanics. In addition, some nonprofit organizations like Common Sense Media provide in-depth reviews and analyses of games beyond age-appropriateness, considering the educational value, positive messages, and the potential for fostering empathy and social skills. To our knowledge, there is currently no widespread system mirroring PEGI or ESRB that specifically endorses games for positive content like empathy, inclusion, or cooperation (Felini, 2015; Lučić et al., 2022).

However, regulation measures are also likely to be met with a critical eye by the gaming community, with some viewing regulation measures as interference. Therefore, these measures should be made in collaboration with influencers in the gaming community to be more deliverable. It is also important to note that while soft regulation can be effective in promoting diversity and inclusion, it may not be sufficient for addressing the most severe cases of online harassment. In such situations, traditional legal measures might be necessary to ensure the safety and well-being of players. Therefore, balancing soft regulation and legal intervention can be crucial to maintaining the gaming community’s trust and cooperation in fostering a more inclusive environment.

### 5.4 Incentivizing investors to support diversity and inclusion in gaming

#### 5.4.1 Importance of investor influence

With the global gaming market surpassing a staggering $365 billions (Straits Research, 2024), investors hold increasing power to influence the industry’s direction and priorities. Investors can influence gaming companies in many ways, via investment decisions, shareholder activism, and engagement with management and sometimes production teams (Tschang, 2007). This is particularly relevant when examining the problems and solutions related to diversity, inclusion, and representation (Gray and Leonard, 2018).

In recent years, there has been a growing recognition of the importance of corporate social responsibility and diversity initiatives, with investors increasingly seeking to align their investments with their values and social impact goals (Dyck et al., 2019). Studies show a positive relationship between a company’s inclusion in diversity rankings and investor returns (McMillan-Capehart et al., 2010), suggesting that the market recognizes and rewards companies that prioritize diversity. The growing body of research that demonstrates the business case for diversity and inclusion underscores the importance of investor influence in shaping the gaming industry. Organizations with diverse teams and inclusive workplace cultures consistently outperform homogeneous counterparts across key metrics of innovation, resilience, and social impact (Duchek et al., 2020; Herring, 2009). The financial benefits prove equally compelling—companies embracing racial and gender diversity achieve superior sales performance, broader customer reach, expanded market presence, and enhanced profitability compared to less diverse companies (Herring, 2009). These findings are crucial for the gaming industry, which is rapidly evolving and increasingly reliant on innovation.

In addition to the immediate business benefits, companies that adhere to diversity and inclusion principles can establish a robust player base for the long term. Conversely, companies that fail to address issues of discrimination, harassment, and toxicity within their games and communities risk alienating players and suffering reputational damage. Studies show that customer experience is essential in enhancing customer loyalty and overall brand value (Biedenbach and Marell, 2010). Indeed, Msosa (2022) shows that ethical practices, including inclusivity, directly contribute to higher customer loyalty in the gaming industry.

These findings highlight the importance of diversity and inclusion not just as ethical imperatives but as strategic business advantages that generate significant financial returns and create positive societal change. Such insights are particularly relevant to the gaming industry, which thrives on creativity and broad market appeal. Therefore, strong arguments can be made towards investors: by choosing to invest in companies that prioritize diversity and inclusion and using their shareholder rights to push for greater accountability and transparency, investors can not only benefit social justice, but their own interests as well (Duchek et al., 2020; Herring, 2009).

#### 5.4.2 Strategies for incentivizing investors to support diversity and inclusion in gaming

Effectively incentivizing investors to support diversity and inclusion in gaming requires a comprehensive approach combining metrics, collaboration, and policy. First, clear and standardized metrics for evaluating gaming companies’ diversity and inclusion efforts can guide investors to favor companies with better diversity scores, especially given diversity’s relationship with better performance and reduced risks (Okatta et al., 2024; Olusanya, 2023).

Another key strategy is creating and supporting coalitions and initiatives specifically promoting diversity and inclusion in games and the gaming community. These coalitions leverage investors’ influence via investor-led campaigns. For example, Invest Ahead (formerly the Thirty Percent Coalition) is an initiative that focuses on increasing diversity on corporate boards and in senior leadership across the United States (Invest Ahead, 2026). Founded in 2011, it includes a wide range of members such as institutional investors, state treasurers, private equity firms, corporate leaders, and advocacy groups. The coalition acts as a powerful alliance advocating for diversity through various means, including legislative and regulatory advocacy and engagement with companies to promote diversity.

Finally, public policy, such as offering tax incentives or government support to gaming companies that demonstrate a commitment to diversity and inclusion, can also motivate investors to support inclusive gaming ventures. This can be taken a step further, by having regulatory bodies incorporate inclusion and diversity standards as necessary requirements for their licensing and approval processes.

### 5.5 Fostering unity and inclusivity through community-led initiatives

While gaming companies, policymakers, and investors play a crucial role in fostering inclusion gaming culture from the top-down, community-led initiatives play a pivotal role in shaping the culture from the bottom-up. Gamers and influencers within each game have the power to establish and maintain safe online spaces by adhering to clear guidelines that negate social exclusion practices and provide the support needed, such as in forms of mentorship. As discussed above, by creating and moderating these spaces, influential community members can significantly contribute to fostering a sense of belonging and inclusivity. Furthermore, prominent figures in the gaming community have the potential to extend their impact beyond individual games by establishing cross-game groups and initiatives. These broader communities can provide a platform for marginalized gamers to connect across different gaming contexts, share resources and strategies for navigating challenging environments, and advocate for change on a larger scale.

Leading gamers and content creators, such as popular streamers and YouTubers, have a particularly influential role. By using their platforms to highlight and celebrate inclusive games, features, and initiatives, they can significantly raise awareness and promote diversity within the gaming industry. They can also educate their audiences through their content, by highlighting the importance of inclusion and diversity values, sharing personal stories, and encouraging their fans to support and participate in community efforts (Gandolfi et al., 2023).

A key component of educational initiatives is raising awareness about the importance of inclusive language and communication and providing the necessary skills and strategies for respectful communication. This can include providing information about the ways in which certain words, phrases, and communication styles can be harmful or exclusionary to marginalized groups, as well as offering guidance on more inclusive alternatives (Sathy and Hogan, 2019).

Individual members of the community can also play their part, by actively reporting discriminatory or exclusive behavior, engaging in positive interactions with other gamers (especially those from marginalized groups), and championing the achievements of inclusion and diversity in the community. Another critical way in which community members can support inclusivity efforts is by vocally endorsing and promoting diversity and inclusion initiatives undertaken by gaming companies and industry leaders. By actively engaging in discussions about the importance of inclusivity and providing positive feedback on inclusive games and features, community members can raise awareness and incentivize the industry to prioritize these issues. As the basis of the gaming community, everyday players who take part in community initiatives, encourage and assist new players, exhibit inclusive behavior, and actively seek out and amplify underrepresented voices, might have the biggest bottom-up impact in this domain.

Finally, content creators in gaming communities (e.g., on Reddit, Discord, Twitch, etc.) can lead by example by cultivating inclusive and welcoming environments within their own communities and online spaces. This can involve setting clear expectations and guidelines for respectful behavior, actively moderating comments and interactions to address any instances of discrimination or hate speech, and using their influence to amplify and support the voices and contributions of marginalized gamers within their communities.

### 5.6 Harnessing the power of technological tools

Data-driven technologies offer powerful solutions for creating more inclusive gaming communities by providing unbiased insights into community dynamics and identifying specific areas needing attention, while also allowing for more innovative solutions. Natural language processing represents a particularly impactful application, automatically detecting hate speech and toxic behavior as it occurs (Sanghvi et al., 2024), thus enabling swift moderation and stopping the escalation of said behavior. Additionally, algorithms can form reputation systems and flag specific players who demonstrate high social exclusion toward others. This, in turn, can be used to create educational prompts and provide information, raising players’ awareness of the importance of inclusivity and urging them to change their ways.

In addition, algorithms can also be used to analyze broader patterns and trends related to inclusivity within gaming communities. By collecting and examining data on player interactions, communication, and behavior across various gaming platforms and contexts, researchers can gain valuable insights into the prevalence and impact of exclusionary practices and the effectiveness of strategies and interventions for promoting inclusivity. This data-driven approach can inform the development of more targeted and evidence-based solutions, as well as help track progress and measure the success of inclusivity initiatives over time.

Finally, to ensure the transparency and accountability of these algorithmic tools, it is crucial to enable external research bodies and institutions to examine and evaluate their performance. Gaming companies can collaborate with these independent organizations by providing access to their algorithms and datasets, allowing for rigorous testing and evaluation while maintaining the confidentiality and anonymity of their users.

### 5.7 The critical role of research

The preceding sections highlighted the multiple ways in which the various immediate stakeholders within the gaming domain can improve inclusion and diversity efforts. In addition to these more obvious parties, there exist other external ones who can also have a great effect on creating a more inclusive and diverse gaming domain, namely, research bodies. By employing rigorous methodologies, collecting and analyzing data, and engaging providing data- and theory-driven solutions, academia and industry-led research has the potential to significantly contribute to the success and long-term sustainability of these efforts. From identifying the root causes of marginalization to evaluating the effectiveness of existing interventions, research can play a pivotal role in informing and guiding the efforts of all stakeholders involved in creating a more inclusive gaming culture.

In the following sections, the current article delves deeper into the specific ways research can contribute to promoting inclusion and diversity within gaming communities. Specifically, we will explore how research can: evaluate the effectiveness of inclusivity initiatives; monitor emerging trends and challenges; gather player feedback and insights; facilitate collaboration and knowledge sharing; and inform policy and regulatory frameworks.

#### 5.7.1 Evaluating the effectiveness of inclusivity initiatives

Measuring the success of inclusivity initiatives requires systematic evaluation to determine whether these efforts create meaningful change. Researchers can apply diverse methodologies to examine these initiatives comprehensively. Longitudinal studies track changes over extended periods (Schnepel et al., 2020), while controlled experiments isolate specific variables (den Van Berg et al., 2020). Surveys capture broad community perspectives (Nelson Laird, 2011), and in-depth interviews reveal nuanced experiences and insights (Zubiri-Esnaola et al., 2020). Through careful data analysis of these various approaches, researchers can identify both intended outcomes and unexpected consequences. This evidence-based evaluation empowers stakeholders to make strategic decisions about resource allocation, program refinement, and expansion of successful interventions.

For instance, researchers can examine the impact of diversity and inclusion training programs on the attitudes and behaviors of game developers and community managers. Similarly, researchers can evaluate the implementation and outcomes of codes of conduct and reporting mechanisms within gaming communities, providing insights into their ability to prevent and address instances of harassment, discrimination, and toxicity. Moreover, research can help to identify best practices and success stories within the gaming industry, highlighting initiatives that have demonstrated significant positive impact on inclusion and diversity. Consequently, researchers can provide valuable lessons and guidelines for other stakeholders seeking to implement similar programs within their own communities and organizations.

Some examples of such evaluation-oriented infrastructures already exist in online gaming. A prominent case is League of Legends’ former “Tribunal” system, which combined player reporting with crowdsourced case review to adjudicate toxic behavior. This system has been studied using large-scale behavioral data and provides a concrete example of how platform governance mechanisms can generate evaluable evidence on community moderation, sanctions, and behavioral norms (Blackburn and Kwak, 2014; Kwak et al., 2015). Subsequent research has also examined Riot Games’ later shift toward more automated governance, highlighting how moderation systems themselves can become objects of empirical evaluation and redesign (Kou and Gui, 2017).

#### 5.7.2 Monitoring emerging trends and challenges

The gaming landscape is constantly evolving, shaped by technological advancements, shifting cultural norms, and emerging social dynamics (Valentine and Jensen, 2016). Staying up-to-date and being able to predict these trends can aid the various stakeholders of the gaming industry in adapting its strategies to remain relevant and effective. By staying attuned to these developments, the gaming ecosystem can proactively address potential inclusivity issues and adapt its strategies to remain relevant and effective. Researchers can employ various methods to track and analyze these emerging trends, including social media analysis (Singh et al., 2019), online ethnography (Gandolfi and Gandolfi, 2021), and data mining (Ekiciler et al., 2022). Through investigation of player discussions, interactions, and behaviors across various platforms and communities, researchers can detect trends and changes in real-time discourse, enabling stakeholders to tackle emerging obstacles and modify their approaches proactively.

Recent developments in the broader virtual ecosystem underscore why such monitoring must extend beyond “traditional” online multiplayer settings. In particular, virtual reality and metaverse-adjacent platforms introduce new affordances that can intensify or transform harms (e.g., embodied and spatialized harassment), while also catalyzing new forms of community governance and safety design. Empirical and conceptual work has documented harassment and safety risks in VR contexts and emphasizes the lack of standardized or universally effective safety measures across VR applications (e.g., blocking/muting, personal boundaries), suggesting an ongoing need to monitor how harms evolve alongside platform affordances and countermeasures (Ortiz, 2022). In another example, researchers can track new forms of harassment and discrimination in gaming communities, including the deployment of deepfakes or AI-generated content targeting marginalized players, while developing countermeasures against these novel challenges.

Furthermore, researchers can provide forward-looking insights and recommendations to stakeholders by analyzing the intersection of gaming with broader societal issues, such as the rise of online extremism or the increasing importance of representation in media. This proactive approach allows the gaming industry to stay ahead of the curve and develop strategies to address potential challenges before they become entrenched within gaming communities.

#### 5.7.3 Gathering player feedback and insights

Inclusive gaming experiences cannot be achieved without actively seeking and incorporating the perspectives, feedback, and insights of marginalized players themselves. By directly engaging with gamers from diverse backgrounds and experiences, researchers can gain valuable firsthand knowledge about the challenges, needs, and aspirations of marginalized groups within the gaming ecosystem.

To gather player feedback researchers can employ a variety of methods, including surveys, focus groups, interviews, and participatory action research. By creating safe and inclusive spaces for gamers to share their stories and perspectives, researchers can amplify the voices of marginalized communities and ensure that their experiences are central to the development of inclusive strategies and initiatives. For instance, researchers can engage with women gamers, and gamers from underrepresented racial and ethnic backgrounds to examine their experiences of representation and inclusion within gaming content and communities. By gathering feedback on the portrayal of diverse characters, narratives, and cultural elements within games, researchers can provide guidance to game developers on how to create more authentic and respectful representations of marginalized groups. Additionally, his feedback can inform the creation of targeted support networks and the implementation of policies and practices that foster a sense of belonging and empowerment for all gamers.

By gathering player feedback and insights through a variety of research methods, the gaming ecosystem can ensure that inclusivity efforts are grounded in the lived experiences and aspirations of marginalized communities, fostering a sense of ownership, representation, and empowerment for all players.

#### 5.7.4 Facilitating collaboration and knowledge dissemination

Research can play a vital role in facilitating collaboration and knowledge sharing among the gaming ecosystem’s various stakeholders. One way to facilitate collaboration and disseminate knowledge is by organizing conferences, workshops, and symposia that bring together game developers, community managers, policymakers, and academic experts to share their experiences, insights, and best practices related to inclusive gaming initiatives. Moreover, researchers can facilitate knowledge sharing by creating open-access repositories or databases that house research papers, case studies, guidelines, toolkits, educational materials, and other resources related to inclusivity in gaming. These repositories can foster collaboration by allowing stakeholders to build upon each other’s work, accelerating progress and innovation.

Finally, research programs can foster a two-way collaboration between the researchers and the community, allowing for a co-creation of solutions and promoting a sense of collaboration. These partnerships can take various forms, such as community-based participatory research, where researchers and community members collaborate throughout the research process, from identifying research questions to interpreting and disseminating findings (Collins et al., 2018). By not only studying them, but also working with marginalized gamers, researchers can get a better understanding of their experiences and challenges, while the gamers themselves benefit from the expertise and resources of the research institution, thus empowering them to address their challenges and contribute to their solution.

Encouragingly, there are already concrete collaboration models that explicitly center disenfranchised player groups in research and development. For example, the AbleGamers Player Panels program was created through a collaboration between the University of York and the AbleGamers charity to provide a systematic means for players with disabilities to have a voice in digital games research and development, and to facilitate matchmaking between disabled players and organizations (including researchers and developers) seeking to conduct user research or game testing (Beeston et al., 2018). Importantly, the program itself was iteratively developed with input from players with disabilities and is designed to lower barriers for inclusion by making it easier for studios and researchers to recruit appropriate participants and incorporate disability-informed feedback into design and evaluation cycles.

In summary, research can play a vital role in informing industry, community, and regulatory guidelines and frameworks aimed at promoting inclusivity and addressing discriminatory practices within the gaming industry. By providing empirical evidence and data-driven insights, research can guide game studios in creating more inclusive games and work atmospheres, and guide policymakers and regulatory bodies in crafting effective guidelines and standards that balance the interests of various stakeholders while upholding the principles of diversity, equity, and inclusion.

### 5.8 A word on the cross-cultural applicability of inclusion norms

As a final consideration, we note an important limitation regarding the scope and transferability of the recommendations proposed in Sections 5.1–5.6. These interventions should not be treated as universally transferable in a one-size-fits-all form. Game cultures are not homogeneous across regions, and cross-cultural game studies explicitly document differences in gaming practices, values, markets, and player-game interactions across national and regional contexts (Sato, 2024). Related work on digital game localization and culturalization likewise shows that developers’ assumptions can unintentionally reflect their own cultural values, and that interventions or design choices that are effective in one context may be poorly received, or even counterproductive, if transplanted without adaptation to another (Pyae, 2018).

Accordingly, we frame the recommendations in Sections 5.1–5.7 as adaptable principles rather than fixed templates. Their implementation should be informed by local stakeholders (including regional developers, community leaders, moderators, creators, and players from marginalized groups), and iteratively tailored to local norms, languages, and platform practices. This approach is consistent with broader literature on cultural adaptation and participatory design, which emphasizes that interventions are more acceptable and effective when they are adapted to local meanings, values, and contexts rather than imposed externally (Perera et al., 2020; Van der Velden and Mörtberg, 2015). In practical terms, this means prioritizing local co-design, pilot testing, and context-specific evaluation while maintaining core goals such as reducing harassment, expanding access, and strengthening players’ sense of safety and belonging.

This point may be especially relevant in regions where gaming cultures, genre conventions, and character norms differ substantially from those most often discussed in Anglophone scholarship. For example, research on Japanese players highlights locally specific preferences and interpretive frameworks (e.g., “gap-moe”) in how players evaluate game characters, underscoring the importance of grounding inclusion-oriented interventions in local gaming cultures rather than assuming cross-regional equivalence (Shibuya et al., 2019).

## 6 Conclusion: the way forward for diversity and inclusion in gaming

Our lives have become inseparable from digital technology. Whether we are working, learning, seeking entertainment, or connecting with others, online platforms now define how we experience and interact with the world around us. This transformation runs especially deep for young people who have never known life without smartphones, social media, and constant connectivity. For them, digital spaces aren’t just tools, they are natural extensions of reality where relationships form, identities develop, and communities thrive. As individuals spend more time in digital and social online spheres, these spaces have become powerful agents in shaping communication patterns, social norms, and cultural values. Members absorb these digital cultures through daily participation, gradually adopting new ways of thinking, communicating, and relating to others. This process of cultural transmission and normalization within online communities can profoundly impact individuals’ worldviews, attitudes, and behaviors, both within and beyond the digital realm.

As the gaming community and industry continue to grow, the importance of balancing inclusion and diversity in the culture of gaming communities will only become more critical. Fostering inclusiveness in gaming requires a collaborative multi-party approach involving industry leaders, regulators, investors, community figures, and individual players. Moreover, ongoing monitoring and adaptation are essential, as inclusivity is a continuous process addressing evolving challenges. Additionally, as the digital world continues to evolve at increasing speeds, research plays an essential role in providing the knowledge, insights, and evidence-based solutions needed to keep up with this pace of cultural evolution. In this article, we suggested metrics to focus the policies and initiatives that promote diversity and inclusion at all levels (see Table 2). By leveraging the power of research, the gaming ecosystem can continuously adapt and refine its strategies, ensuring that they remain responsive to the evolving needs and challenges faced by marginalized players and effectively promoting a more diverse and inclusive environment in the culture of gaming communities.

**Table 2:** Solutions and recommendations.

Stakeholder: problem to address	Industry		Gaming community			Regulation and policy
	Gaming companies developers, publishers	Investors	Influencers, streamers, content creators	Community leaders, moderators, advocacy groups	Individual players	Regulators, policymakers
Stereotypical representations and oversexualization of women characters	Prioritize diverse, multidimensional women character designs; implement guidelines for inclusive representation	Prioritize investments in companies with inclusive practices and diverse teams	Highlight and celebrate games with authentic women character portrayals	Establish community guidelines against harmful stereotypes; foster inclusive discussions	Provide feedback on problematic representations; support inclusive games	Develop content rating systems and guidelines addressing stereotypical representations
Lack of racial/ethnic diversity in characters and narratives	Incorporate diverse perspectives in creative processes; collaborate with underrepresented writers/consultants	Engage with companies on diversity and inclusion efforts	Amplify games with diverse characters and narratives	Create safe spaces for underrepresented communities; facilitate feedback and collaboration with developers	Demand and support games with authentic representation	Incentivize diverse representation through policies and initiatives
Homophobic language, anti-LGBTQ+ attitudes, heteronormativity	Include LGBTQ+ representation and narratives; challenge heteronormative assumptions	Advocate for LGBTQ+ inclusion within gaming companies	Cultivate inclusive spaces; promote LGBTQ+ content creators	Implement clear guidelines against hate speech and discrimination; provide resources and support for LGBTQ+ gamers	Challenge homophobic language and behaviors; support LGBTQ+ gamers	Develop regulations addressing online hate speech and discrimination
Discrimination, harassment, and toxicity	Robust community moderation systems; algorithms to detect hate speech and toxic behavior	Pressure companies to address harassment and toxicity within their platforms	Model inclusive behavior; report instances of harassment	Establish effective reporting mechanisms; take appropriate actions against offenders	Call out and report toxic behavior; promote positivity	Enforce laws and regulations related to online harassment and discrimination
Lack of awareness of marginalized gamers’ experiences	Educational campaigns highlighting marginalized experiences	Fund research and advocacy efforts related to inclusivity	Use platforms to educate audiences; promote inclusive values	Organize workshops, panels, and events to raise awareness; collaborate with advocacy groups	Seek to understand diverse perspectives; challenge biases	Support educational initiatives and awareness campaigns
Algorithmic assortative matching limiting diversity	Incorporate diversity as a factor in matchmaking algorithms; enable research access	Invest in companies with ethical and inclusive AI practices	–	Advocate for diverse matchmaking practices; provide feedback and insights	–	Develop guidelines and regulations for algorithmic fairness and transparency
Accessibility barriers for disabled gamers	Prioritize inclusive design principles and accessibility features; collaborate with disability advocacy groups	Support companies with inclusive and accessible product offerings	Highlight accessibility features; support inclusive developers	Facilitate collaboration between developers and disability communities	Provide feedback on accessibility needs	Establish accessibility standards and guidelines for gaming platforms
Lack of workforce diversity	Inclusive hiring and retention practices; dedicated diversity and inclusion initiatives	Engage with companies on diversity and inclusion initiatives	–	Partner with industry to support diverse talent pipelines	–	Develop policies and incentives to promote workforce diversity
Marginalization of intersectional identities	Intersectional approach to character design and narratives; collaborate with intersectional organizations	Support organizations and initiatives focused on intersectionality	Amplify intersectional voices and experiences	Create safe spaces for individuals with intersectional identities; advocate for their needs	Recognize complexities of intersectional oppression	Develop guidelines and resources for addressing intersectional issues
Lack of transparency and accountability	Robust reporting on diversity efforts; independent audits	Prioritize investments in transparent and accountable companies	–`	Advocate for transparency and accountability measures; provide community feedback	–	Mandate reporting and disclosure requirements for diversity and inclusion efforts

## Data Availability

The original contributions presented in the study are included in the article/Supplementary material, further inquiries can be directed to the corresponding author.

## Footnotes

1 See (Dewey, 2014) for a short summary of the incident.

## References

[ref1] Adachi P. J. Hodson G. Willoughby T. Blank C. Ha A. (2016). From outgroups to allied forces: effect of intergroup cooperation in violent and nonviolent video games on boosting favorable outgroup attitudes. J. Exp. Psychol. Gen. 145, 259–265. doi: 10.1037/xge0000145, 26881988

[ref9014] Adinolf S. Turkay S. (2018). “Toxic behaviors in Esports games: player perceptions and coping strategies” in Proceedings of the 2018 Annual Symposium on Computer-Human Interaction in Play Companion Extended Abstracts, 365–372. doi: 10.1145/3270316.3271545

[ref2] AlAfnan M. A. (2025). Language, power, and social dynamics in online gaming: a discourse analysis of toxicity and inclusivity in digital spaces. Stud. Media Communic. 13:62. doi: 10.11114/smc.v13i2.7512

[ref3] Allaire J. C. McLaughlin A. C. Trujillo A. Whitlock L. A. LaPorte L. Gandy M. (2013). Successful aging through digital games: socioemotional differences between older adult gamers and non-gamers. Comput. Human Behav. 29, 1302–1306. doi: 10.1016/j.chb.2013.01.014

[ref4] Allen J. J. Anderson C. A. (2018). Satisfaction and frustration of basic psychological needs in the real world and in video games predict internet gaming disorder scores and well-being. Comput. Human Behav. 84, 220–229. doi: 10.1016/j.chb.2018.02.034

[ref5] Anglada-Pujol O. (2022). Our fans are gonna go crazy when they know we are together. Mediální studia 16, 196–214.

[ref501] Araújo M. C. Façanha A. R. Darin T. G. Sánchez J. Andrade R. M. Viana W. (2017). “Mobile audio games accessibility evaluation for users who are blind” in International Conference on Universal Access in Human-Computer Interaction (Cham: Springer International Publishing), 242–259. doi: 10.1007/978-3-319-58703-518

[ref6] Attaran M. Gunasekaran A. Attaran M. Gunasekaran A. (2019). “Blockchain for gaming,” in Applications of Blockchain Technology in Business: Challenges and Opportunities, Attaran, M., and Gunasekaran, A. eds., Challenges and opportunities. Springer. 85–88. doi: 10.1007/978-3-030-27798-7_12

[ref7] Ayal S. Gino F. Barkan R. Ariely D. (2015). Three principles to REVISE people’s unethical behavior. Perspect. Psychol. Sci. 10, 738–741. doi: 10.1177/1745691615598512, 26581728

[ref8] Babichenko D. Radovic A. Patel R. Hester A. Powell K. Eggers N. . (2023). Evaluating the feasibility of a multiplayer role-playing game as a behavioral health intervention in adolescent patients with chronic physical or mental conditions: protocol for a cohort study. JMIR Res. Protoc. 12:e43987. doi: 10.2196/43987, 37368477 PMC10337425

[ref9] Ballard M. E. Welch K. M. (2017). Virtual warfare: cyberbullying and cyber-victimization in MMOG play. Games Cult. 12, 466–491. doi: 10.1177/1555412015592473

[ref10] Banerjee D. Vajawat B. Varshney P. (2021). Digital gaming interventions: a novel paradigm in mental health? Perspectives from India. Int. Rev. Psychiatry 33, 435–441. doi: 10.1080/09540261.2020.1839392 33210563

[ref11] Barkan R. Ayal S. Ariely D. (2015). Ethical dissonance, justifications, and moral behavior. Curr. Opin. Psychol. 6, 157–161. doi: 10.1016/j.copsyc.2015.08.001

[ref12] Beeston J. Power C. Cairns P. Barlet M. (2018). Accessible Player Experiences (APX): The Players. In International Conference on Computers Helping People with special Needs (pp. 245–253). Cham: Springer International Publishing.

[ref13] Bergstrom K. (2021). Anti-social social gaming: community conflict in a Facebook game. Crit. Stud. Media Commun. 38, 61–74. doi: 10.1080/15295036.2020.1863441

[ref14] Biedenbach G. Marell A. (2010). The impact of customer experience on brand equity in a business-to-business services setting. J. Brand Manag. 17, 446–458. doi: 10.1057/bm.2009.37

[ref15] Birk M. V. Buttlar B. Bowey J. T. Poeller S. Thomson S. C. Baumann N. . (2016) The effects of social exclusion on play experience and hostile cognitions in digital games. In Proceedings of the 2016 CHI Conference on human Factors in Computing Systems (pp. 3007–3019)

[ref16] Blackburn J. Kwak H. (2014) STFU NOOB! Predicting crowdsourced decisions on toxic behavior in online games. In Proceedings of the 23rd International Conference on World wide web (pp. 877–888).

[ref17] Blanco-Fernández V. Moreno J. A. (2023). “Video games were my first safe space”: queer gaming in the animal crossing new horizons LGBTIQA community. Games Cult.:15554120231205638. doi: 10.1177/15554120231205638

[ref18] Blomqvist P. (2022). “Soft and hard governing tools,” in Christopher Ansell and Jacob Torfing eds., Handbook on Theories of Governance, (Cheltenham, UK & Northampton, MA, USA: Edward Elgar Publishing), 285–296.

[ref19] Boroń M. Brzeziński J. Kobusińska A. (2020). P2P matchmaking solution for online games. Peer-to-Peer Netw. Appl. 13, 137–150. doi: 10.1007/s12083-019-00725-3

[ref20] Boudreau K. (2022). Beyond deviance: toxic gaming culture and the potential for positive change. Crit. Stud. Media Commun. 39, 181–190. doi: 10.1080/15295036.2022.2080848

[ref21] Braithwaite A. (2014). Seriously, get out’: feminists on the forums and the war (craft) on women. New Media Soc. 16, 703–718. doi: 10.1177/1461444813489503

[ref22] Braithwaite A. (2016). It’s about ethics in games journalism? Gamergaters and geek masculinity. Soc. Media Soc. 2:2056305116672484. doi: 10.1177/2056305116672484

[ref23] Brehm A. L. (2013). Navigating the feminine in massively multiplayer online games: gender in world of Warcraft. Front. Psychol. 4:903. doi: 10.3389/fpsyg.2013.00903, 24363650 PMC3849516

[ref24] Brenner-Levoy J. (2023). Virtually masculine: queer men’s experiences with harassment in online video games. Sociol. Sport J. 40, 385–398. doi: 10.1123/ssj.2022-0170

[ref9002] Brewer J. Romine M. Taylor T. L. (2020). “Inclusion at scale: deploying a community-driven moderation intervention on twitch” in Proceedings of the 2020 ACM Designing Interactive Systems Conference, 757–769. doi: 10.1145/3357236.3395514

[ref25] Brint S. (2001). Gemeinschaft revisited: a critique and reconstruction of the community concept. Sociol Theory 19, 1–23. doi: 10.1111/0735-2751.00125

[ref26] Brooker R. (2026). Gaming communities in Lethbridge: understanding barriers to participation and pathways to inclusion. J. Int. Stud. 17, 1–34. Available online at: https://jis.athabascau.ca/index.php/jis/article/view/495

[ref9003] Brown J. A. (2016). “Exploring the next generation of older gamers: middle-aged gamers” in Human Aspects of IT for the Aged Population. Healthy and Active Aging. ITAP 2016. eds. Zhou J. Salvendy G., Lecture Notes in Computer Science, vol. 9755 (Cham: Springer). doi: 10.1007/978-3-319-39949-2_30

[ref9004] Brown J. A. (2017). “Digital gaming perceptions among older adult non-gamers” in Human Aspects of IT for the Aged Population. Applications, Services and Contexts. ITAP 2017. eds. Zhou J. Salvendy G., Lecture Notes in Computer Science, vol. 10298 (Cham: Springer). doi: 10.1007/978-3-319-58536-9_18

[ref27] Brown A. M. Moberly L. (2020). “Twitch and participatory cultures,” in Rachel Kowert and Thorsten Quandt eds., The Video Game Debate 2, (New York, NY, USA: Routledge), 53–65.

[ref28] Burger-Helmchen T. Cohendet P. (2011). User communities and social software in the video game industry. Long Range Plan. 44, 317–343. doi: 10.1016/j.lrp.2011.09.003

[ref29] Bustamante A. Chaux E. (2014). Reducing moral disengagement mechanisms: a comparison of two interventions. J. Latino/Lat. Am. Stud. 6, 52–54. doi: 10.18085/llas.6.1.123583644qq115t3

[ref30] Bustos-Ortega M. Carretero-Dios H. Megías J. L. Romero-Sánchez M. (2023). Sexist attitudes in online video gaming: development and validation of the sexism against women gamers scale in Spanish and English. Psychol. Women Q. 47, 320–342. doi: 10.1177/03616843231162837

[ref31] Cary L. A. Axt J. Chasteen A. L. (2020). The interplay of individual differences, norms, and group identification in predicting prejudiced behavior in online video game interactions. J. Appl. Soc. Psychol. 50, 623–637. doi: 10.1111/jasp.12700

[ref32] Cary L. A. Chasteen A. L. (2022). Prejudice norms in online gaming: game context and gamer identification as predictors of the acceptability of prejudice. Psychol. Pop. Media 11:367. doi: 10.1037/ppm0000377

[ref33] Chamberlain E. F. (2022). “Our world is worth fighting for”: gas mask agency, copypasta sit-ins, and the material-discursive practices of the Blitzchung controversy. Comput. Compos. 65:102725. doi: 10.1016/j.compcom.2022.102725

[ref34] Chess S. Shaw A. (2015). A conspiracy of fishes, or, how we learned to stop worrying about# gamergate and embrace hegemonic masculinity. J. Broadcast. Electron. Media 59, 208–220. doi: 10.1080/08838151.2014.999917

[ref5001] Churchill B. C. Xu W. (2016). “The modem nation: a first study on twitch. TV social structure and player/game relationships” in 2016 IEEE International Conferences on Big Data and Cloud Computing (BDCloud), Social Computing and Networking (SocialCom), Sustainable Computing and Communications (SustainCom)(BDCloud-SocialCom-SustainCom) (IEEE), 223–228. doi: 10.1109/BDCloud-SocialCom-SustainCom.2016.43

[ref35] Cimolino G. Askari S. Graham T. C. N. (2021). The role of partial automation in increasing the accessibility of digital games. Proc. ACM Human Comput. Interact. 5, 1–30. doi: 10.1145/347469336644216

[ref36] Clark J. Leavitt A. Williams D. (2015) Online Games, Community Aspects of the International Encyclopedia of Digital Communication and Society. In The International Encyclopedia of Digital Communication and Society, P.H. Ang and R. Mansell eds., (Chichester, West Sussex, UK: Wiley-Blackwell). doi: 10.1002/9781118767771.wbiedcs053

[ref37] Collins S. E. Clifasefi S. L. Stanton J. Straits K. J. Gil-Kashiwabara E. Rodriguez Espinosa P. . (2018). Community-based participatory research (CBPR): towards equitable involvement of community in psychology research. Am. Psychol. 73, 884–898. doi: 10.1037/amp0000167, 29355352 PMC6054913

[ref38] Cook C. L. Karhulahti V. Harrison G. Bowman N. D. (2024). Trolligans: conceptual links between trolling and hooliganism in sports and esports. Commun. Sport 12, 397–418. doi: 10.1177/21674795231153005

[ref39] Cote A. C. (2017). “I can defend myself” women’s strategies for coping with harassment while gaming online. Games Cult. 12, 136–155. doi: 10.1177/1555412015587603

[ref40] Cote A. C. (2018). Writing “gamers” the gendered construction of gamer identity in Nintendo power (1994–1999). Games Cult. 13, 479–503. doi: 10.1177/1555412015624742

[ref41] Crowe N. Watts M. (2014). When i click “ok” I become sassy–I become a girl’. Young people and gender identity: subverting the ‘body’in massively multi-player online role-playing games. Int. J. Adolesc. Youth 19, 217–231. doi: 10.1080/02673843.2012.736868

[ref9006] Crenshaw N. Nardi B. (2015). NPCs as social mediators in massively multiplayer online games. In Proceedings of the AAAI Conference on Artificial Intelligence and Interactive Digital Entertainment (Vol. 11, No., pp. 89–91).

[ref42] Daneels R. Bowman N. D. Possler D. Mekler E. D. (2021). The ‘eudaimonic experience’: a scoping review of the concept in digital games research. Media Commun. 9, 178–190. doi: 10.17645/mac.v9i2.3824

[ref43] Darvin L. Levine J. Keaton A. (2025). Examining the culture of diversity, equity, and inclusion practices within the video game industry through an anti-isms lens. J. Electron. Gaming Esports 3:21. doi: 10.1123/jege.2024-0021

[ref44] Delalleau O. Contal E. Thibodeau-Laufer E. Ferrari R. C. Bengio Y. Zhang F. (2012). Beyond skill rating: advanced matchmaking in ghost recon online. IEEE Trans. Comput. Intell. AI Games 4, 167–177. doi: 10.1109/tciaig.2012.2188833

[ref45] den Van Berg A. C. Giest S. N. Groeneveld S. M. Kraaij W. (2020). Inclusivity in online platforms: recruitment strategies for improving participation of diverse sociodemographic groups. Public Adm. Rev. 80, 989–1000. doi: 10.1111/puar.13215

[ref46] Devereaux T. (2023). The erasure of Asian gamers: the gaming industry as a racialized social structure. Sociation 22, 54–62.

[ref47] Dewey C. (2014). The only guide to Gamergate you will ever need to read. The Washington Post. Available online at: https://www.washingtonpost.com/news/the-intersect/wp/2014/10/14/the-only-guide-to-gamergate-youwill-ever-need-to-read/

[ref48] Di Leo N. Rizzi A. Traetta L. (2025). Stressing out the ‘damsel in distress’: intersectional shifts in women’s representation in video games. Postdigit. Sci. Educ. 8, 113–139. doi: 10.1007/s42438-025-00607-3

[ref49] Dietrich D. R. (2023). Reexamining avatars of whiteness: changes in racial presentation of video game player characters. Sociation 22, 21–31.

[ref50] Duchek S. Raetze S. Scheuch I. (2020). The role of diversity in organizational resilience: a theoretical framework. Bus. Res. 13, 387–423. doi: 10.1007/s40685-019-0084-8

[ref51] Duran C. S. (2017). “You not die yet”: Karenni refugee children's language socialization in a video gaming community. Linguist. Educ. 42, 1–9. doi: 10.1016/j.linged.2017.09.002

[ref52] Dyck A. Lins K. V. Roth L. Wagner H. F. (2019). Do institutional investors drive corporate social responsibility? International evidence. J. Financ. Econ. 131, 693–714. doi: 10.1016/j.jfineco.2018.08.013

[ref53] Ekiciler A. Ahioğlu İ. Yıldırım N. Ajas İ. İ. Kaya T. (2022). The bullying game: sexism based toxic language analysis on online games chat logs by text mining. J. Int. Womens Stud. 24, 1–16. Available online at: https://www.scopus.com/inward/record.uri?eid=2-s2.0-85134998324&partnerID=40&md5=6e6bc81ee9201371bc0a80eb31071f8d

[ref54] Entertainment Software Association (2024). Essential facts 2024. Washington, D.C. USA: Entertainment Software Association (ESA). Available online at: https://www.theesa.com/wp-content/uploads/2024/05/Essential-Facts-2024-FINAL.pdf

[ref9007] Farah Y. A. Dorneich M. C. Gilbert S. B. (2022). Evaluating team metrics in cooperative video games. In Proceedings of the Human Factors and Ergonomics Society Annual Meeting (Vol. 66, No., pp. 70–74). Los Angeles, CA: Sage Publications. https://doi.org/10.1177/1071181322661240

[ref55] Felini D. (2015). Beyond today’s video game rating systems: a critical approach to PEGI and ESRB, and proposed improvements. Games Cult. 10, 106–122. doi: 10.1177/1555412014560192

[ref56] Fisher L. B. Overholser J. C. Ridley J. Braden A. Rosoff C. (2015). From the outside looking in: sense of belonging, depression, and suicide risk. Psychiatry 78, 29–41. doi: 10.1080/00332747.2015.1015867, 26168025

[ref57] Fordyce R. Neale T. Apperley T. H. (2018). “Avatars: addressing racism and racialised address,” in Kishonna L. Gray and David J. Leonard, eds., Woke Gaming: Digital Challenges to Oppression and Social Injustice, (Seattle, WA, USA: University of Washington Press), 231–251.

[ref58] Fox J. Tang W. Y. (2014). Sexism in online video games: the role of conformity to masculine norms and social dominance orientation. Comput. Human Behav. 33, 314–320. doi: 10.1016/j.chb.2013.07.014

[ref59] Fox J. Tang W. Y. (2017). Women’s experiences with general and sexual harassment in online video games: rumination, organizational responsiveness, withdrawal, and coping strategies. New Media Soc. 19, 1290–1307. doi: 10.1177/1461444816635778

[ref60] Frommel J. Johnson D. Mandryk R. L. (2023). How perceived toxicity of gaming communities is associated with social capital, satisfaction of relatedness, and loneliness. Comput. Human Behav. Rep. 10:100302. doi: 10.1016/j.chbr.2023.100302

[ref9008] Frommel J. Sagl V. Depping A. Johanson C. Miller M. Mandryk R. (2020). “Recognizing affiliation: using behavioural traces to predict the quality of social interactions in online games” in Proceedings of the 2020 CHI Conference on Human Factors in Computing Systems. doi: 10.1145/3313831.3376446

[ref61] Gandolfi E. Ferdig R. E. Soyturk I. (2023). Exploring the learning potential of online gaming communities: an application of the game communities of inquiry scale. New Media Soc. 25, 1374–1393. doi: 10.1177/14614448211027171

[ref62] Gandolfi E. Gandolfi S. (2021). Playing across the social zone-animal crossing, gaming communities and connectedness in a time of crisis. Acad. Int. Sci. J. 12, 41–51. doi: 10.7336/academicus.2021.23.03

[ref63] Garcia F. de Almeida Neris V. (2020). A framework for tailorable games: toward inclusive end-user development of inclusive games. Universal Access Inf. Soc. 21, 193–237. doi: 10.1007/s10209-020-00779-8

[ref64] Ghosh A. (2021). Analyzing toxicity in online gaming communities. Turk. J. Comput. Math. Educ. 12, 4448–4455. Available online at: https://turcomat.org/index.php/turkbilmat/article/view/5182

[ref65] Gillin L. E. Signorella M. L. (2024). Attitudes toward sexual orientation and gender identity in online multiplayer gaming spaces. Psychol. Rep. 127, 3066–3088. doi: 10.1177/00332941231153798, 36688329 PMC11529123

[ref66] Giolla Easpaig B. Humphrey H. (2017). “Pitching a virtual woo”: analysing discussion of sexism in online gaming. Feminism Psychol. 27, 553–561. doi: 10.1177/0959353516667400

[ref143] Gottfried J. Sidoti O. (2024). Teens and video games today. Washington, D.C.: Pew Research Center. Available at: https://www.pewresearch.org/wpcontent/uploads/sites/20/2024/05/PI_2024.05.09_Video-Games_REPORT.pdf (Accessed April 4, 2026).

[ref67] Graham S. L. (2019). A wink and a nod: the role of emojis in forming digital communities. Multilingua 38, 377–400. doi: 10.1515/multi-2018-0037

[ref68] Gray K. L. (2012a). Diffusion of innovation theory and xbox live: examining minority gamers’ responses and rate of adoption to changes in xbox live. Bull. Sci. Technol. Soc. 32, 463–470. doi: 10.1177/0270467612469076

[ref69] Gray K. L. (2012b). Intersecting oppressions and online communities: examining the experiences of women of color in Xbox live. Inf. Commun. Soc. 15, 411–428. doi: 10.1080/1369118X.2011.642401

[ref70] Gray K. L. (2018). Gaming out online: Black lesbian identity development and community building in Xbox live. J. Lesbian Stud. 22, 282–296. doi: 10.1080/10894160.2018.1384293, 29166214

[ref71] Gray K. L. Leonard D. J. (2018). Woke Gaming: Digital Challenges to Oppression and Social Injustice. Seattle, WA, USA: University of Washington Press.

[ref72] Haddaway N. R. Grainger M. J. Gray C. T. (2022). Citationchaser: A tool for transparent and efficient forward and backward citation chasing in systematic searching. *Research Synthesis Methods*, 13, 533–545. doi: 10.1002/jrsm.1563 35472127

[ref73] Hagerty B. M. Williams A. (1999). The effects of sense of belonging, social support, conflict, and loneliness on depression. Nurs. Res. 48, 215–219. doi: 10.1097/00006199-199907000-00004, 10414684

[ref74] Hanus M. D. Dickinson T. M. (2019). The (faulty) assumption that male players prefer male characters: how character desirability and likability influence video game purchase intentions and enjoyment. Psychol. Pop. Media Cult. 8, 395–401. doi: 10.1037/ppm0000191

[ref75] Harvey A. (2014). Twine’s revolution: democratization, depoliticization, and the queering of game design. Games Art Media Enterta. 1, 95–107.

[ref76] Hasei J. Matsumoto Y. Kawai H. Okahisa Y. Takaki M. Ozaki T. (2025). Metaverse support groups for LGBTQ+ youth: an observational study on safety, self-expression, and early intervention. J. Metaverse 5, 156–167. doi: 10.57019/jmv.1639701

[ref77] Hayday E. J. Collison H. (2020). Exploring the contested notion of social inclusion and gender inclusivity within esport spaces. Soc. Inclus. 8, 197–208. doi: 10.17645/si.v8i3.2755

[ref78] Herring C. (2009). Does diversity pay?: race, gender, and the business case for diversity. Am. Sociol. Rev. 74, 208–224. doi: 10.1177/000312240907400203

[ref9009] Herodotou C. (2010). Social praxis within and around online gaming: the case of world of Warcraft. Third IEEE International Conference on Digital Game and Intelligent Toy Enhanced Learning, Kaohsiung, Taiwan 2010, 10–22. doi: 10.1109/DIGITEL.2010.31

[ref79] Hou J. (2011). Uses and Gratifications of Social Games: Blending Social Networking and Game Play. First Monday, 16. Available online at: https://firstmonday.org/ojs/index.php/fm/article/download/3517/3020

[ref9010] Horton E. Johnson D. Mitchell J. (2016). “Finding and building connections: moving beyond skill-based matchmaking in videogames” in Proceedings of the 28th Australian Conference on Computer-Human Interaction, 656–658. doi: 10.1145/3010915.3011857

[ref9011] Konert J. Göbel S. Steinmetz R. (2014). “Video game personalization via social media participation” in International Conference on Serious Games (Cham: Springer International Publishing), 35–46.

[ref80] Hussain Z. Griffiths M. D. (2009). The attitudes, feelings, and experiences of online gamers: a qualitative analysis. Cyberpsychol. Behav. 12, 747–753. doi: 10.1089/cpb.2009.0059, 19788376

[ref81] Invest Ahead (2026). About us- Invest Ahead. Available online at: https://www.invest-ahead.org/about

[ref82] Jagayat A. Choma B. L. (2021). Cyber-aggression towards women: measurement and psychological predictors in gaming communities. Comput. Hum. Behav. 120:106753. doi: 10.1016/j.chb.2021.106753

[ref83] Jansz J. Martens L. (2005). Gaming at a LAN event: the social context of playing video games. New Media Soc. 7, 333–355. doi: 10.1177/1461444805052280

[ref84] Jerrett A. Howell P. M. (2022). Values throughout the game space. Proceedings of the ACM on Human-Computer Interaction 6, 1–27.

[ref85] Jones S. S. Y. Harrisson A. Pedraça S. Marchessault-Brown J. Williams D. Consalvo M. (2025). The virtual census 2.0: a continued investigation on the representations of gender, race, and age in videogames. New Media Soc.:14614448251336427. doi: 10.1177/14614448251336427

[ref86] Jubran H. S. Y. Karimi F. Al-Jameel B. J. K. (2025). Online hate speech in the gaming community: a critical discourse analysis. J. New Trends English Lang. Learn. 4, 1–11. doi: 10.57647/jntell.2025.0403.16

[ref87] Jung C. W. (2020). Role of gamers’ communicative ecology on game community involvement and self-identification of gamer. Comput. Hum. Behav. 104:106164. doi: 10.1016/j.chb.2019.106164

[ref88] Kang H. Suh C. Kim H. K. (2024). Match experiences affect interest: impacts of matchmaking and performance on churn in a competitive game. Heliyon 10:e24891. doi: 10.1016/j.heliyon.2024.e24891, 38318006 PMC10839887

[ref89] Kaye L. K. Gresty C. E. Stubbs-Ennis N. (2017). Exploring stereotypical perceptions of female players in digital gaming contexts. Cyberpsychol. Behav. Soc. Netw. 20, 740–745. doi: 10.1089/cyber.2017.0294, 29211508

[ref90] Kelly D. Nic Giolla Easpaig B. Castillo P. (2023). You game like a girl’: perceptions of gender and competence in gaming. Games Cult. 18, 62–78. doi: 10.1177/15554120221077730

[ref91] Kim D. Russworm T. M. Vaughan C. Adair C. Paredes V. Cowan T. L. (2018). Race, gender, and the technological turn: a roundtable on digitizing revolution. Frontiers 39, 149–177. Available online at: https://www.jstor.org/stable/10.5250/fronjwomestud.39.1.0149

[ref92] Király O. Griffiths M. D. King D. L. Lee H. Lee S. Bányai F. . (2018). Policy responses to problematic video game use: a systematic review of current measures and future possibilities. J. Behav. Addict. 7, 503–517. doi: 10.1556/2006.6.2017.050, 28859487 PMC6426392

[ref93] Komaç G. Çağıltay K. (2021). “Raising awareness through games: the influence of a trolling game on perception of toxic behavior,” in Game Design Education: Proceedings of PUDCAD 2020, (Cham: Springer International Publishing), 143–154.

[ref94] Kordyaka B. Jahn K. Niehaves B. (2020). Towards a unified theory of toxic behavior in video games. Internet Res. 30, 1081–1102. doi: 10.1108/intr-08-2019-0343

[ref95] Kou Y. Gui X. (2017) When code governs community. In eLibrary A. (Ed.), Proceedings of the 50th hawaii International Conference on system Sciences.

[ref96] Koulouris J. Jeffery Z. Best J. O'neill E. Lutteroth C. (2020). Me vs. super (wo) man: effects of customization and identification in a VR Exergame. In Proceedings of the 2020 CHI Conference on human Factors in Computing Systems (pp. 1–17).

[ref97] Kowert R. (2020). Dark participation in games. Front. Psychol. 11:598947. doi: 10.3389/fpsyg.2020.598947, 33244307 PMC7683775

[ref98] Kowert R. Festl R. Quandt T. (2014). Unpopular, overweight, and socially inept: reconsidering the stereotype of online gamers. Cyberpsychol. Behav. Soc. Netw. 17, 141–146. doi: 10.1089/cyber.2013.0118, 24053382

[ref99] Kowert R. Martel A. Swann W. B. (2022). Not just a game: identity fusion and extremism in gaming cultures. Front. Commun. 7:1007128. doi: 10.3389/fcomm.2022.1007128

[ref100] Kraus S. Breier M. Dasí-Rodríguez S. (2020). The art of crafting a systematic literature review in entrepreneurship research. Int. Entrep. Manag. J. 16, 1023–1042. doi: 10.1007/s11365-020-00635-4

[ref101] Krobová T. Moravec O. Švelch J. (2015). Dressing commander Shepard in pink: queer playing in a heteronormative game culture. Cyberpsychol. J. Psychosoc. Res. Cyberspace 9:3. doi: 10.5817/CP2015-3-3

[ref102] Kuipers F. Märtens M. van der Hoeven E. Iosup A. (2018). “The power of social features in online gaming,” In: K. Lakkaraju, G. Sukthankar, and R. T. Wigand (Eds.), Social Interactions in Virtual Worlds: An Interdisciplinary Perspective, 313–336.

[ref103] Kukshinov E. Shaw A. (2022). Playing with privilege: examining demographics in choosing player-characters in video games. Psychol. Pop. Media 11:90. doi: 10.1037/ppm0000378

[ref104] Kwak H. Blackburn J. Han S. (2015) Exploring cyberbullying and other toxic behavior in team competition online games. In Proceedings of the 33rd Annual ACM Conference on human Factors in Computing Systems (pp. 3739–3748)

[ref105] la De Torre-Sierra A. M. Guichot-Reina V. (2025). Women in video games: an analysis of the biased representation of female characters in current video games. Sex. Cult. 29, 532–560. doi: 10.1007/s12119-024-10286-0

[ref106] Lavigne C. (2015). ‘She’s a soldier, not a model’: feminism, FemShep and the mass effect 3 vote. J. Gaming Virtual Worlds 7, 317–329. doi: 10.1386/jgvw.7.3.317

[ref107] Le Hénaff B. Michinov N. Le Bohec O. Delaval M. (2015). Social gaming is inSIDE: impact of anonymity and group identity on performance in a team game-based learning environment. Comput. Educ. 82, 84–95. doi: 10.1016/j.compedu.2014.11.002

[ref108] Lewis Z. H. Swartz M. C. Lyons E. J. (2016). What's the point?: a review of reward systems implemented in gamification interventions. Games Health J. 5, 93–99. doi: 10.1089/g4h.2015.0078, 26812253

[ref109] Lin J. Y. Wang E. S. Kao L. L. Cheng J. M. (2007). A study of the perceived recognition affecting the adoption of innovation with respect to the online game in Taiwan. Cyberpsychol. Behav. 10, 813–816. doi: 10.1089/cpb.2007.9949, 18085969

[ref110] López Vargas K. Runge J. Zhang R. (2022). Algorithmic assortative matching on a digital social medium. Inf. Syst. Res. 33, 1138–1156. doi: 10.1287/isre.2022.1135

[ref111] Lucero L. (2017). Safe spaces in online places: social media and LGBTQ youth. Multicult. Educ. Rev. 9, 117–128. doi: 10.1080/2005615x.2017.1313482

[ref112] Lučić A. Uzelac M. Vidović N. (2022). Critical review of children consumer protection national policies. J. Macromark. 42, 510–532. doi: 10.1177/02761467221111159

[ref113] Marston H. R. Freeman S. Bishop K. A. Beech C. L. (2016). A scoping review of digital gaming research involving older adults aged 85 and older. Games Health J. 5, 157–174. doi: 10.1089/g4h.2015.0087, 27096726

[ref114] Martínez M. H. (2020). Feminist cyber-resistance to digital violence: surviving gamergate. Debats Rev. Cult. Poder Societat 5, 287–302. doi: 10.28939/iam.debats-en.2020-17

[ref115] Mäyrä F. (2015). Exploring gaming communities. In R. Kowert and T. Quandt (Eds.), The video game debate: Unravelling the physical, social, and psychological effects of video games. New York: Routledge. 153–175. doi: 10.4324/9781315736495

[ref116] McKinnon-Crowley S. (2020). Fighting gendered battles: on being a woman in a contemporary gaming community. J. Contemp. Ethnogr. 49, 118–142. doi: 10.1177/0891241619864405

[ref117] McLean L. Griffiths M. D. (2019). Female gamers’ experience of online harassment and social support in online gaming: a qualitative study. Int. J. Ment. Health Addict. 17, 970–994. doi: 10.1007/s11469-018-9962-0

[ref118] McMillan-Capehart A. Aaron J. R. Cline B. N. (2010). Investor reactions to diversity reputation signals. Corp. Reput. Rev. 13, 184–197. doi: 10.1057/crr.2010.20

[ref119] Meric E. (2026). “From competition to conflict: hate speech in multiplayer online Battle arena games (MOBAs),” In: M. N. Erdem and Ö. A. Kuş (Eds.), Critical Perspectives on Digital Culture and Gaming, (Hershey, PA: IGI Global Scientific Publishing), 159–190. doi: 10.4018/979-8-3373-6192-5

[ref120] Michalik L. (2015). Stretching the code: sexual performances and online gaming economies. Liminalities 11:1.

[ref121] Molyneux L. Vasudevan K. de Gil Zúñiga H. (2015). Gaming social capital: exploring civic value in multiplayer video games. J. Comput.-Mediat. Commun. 20, 381–399. doi: 10.1111/jcc4.12123

[ref122] Moore C. (2015). Moral disengagement. Curr. Opin. Psychol. 6, 199–204. doi: 10.1016/j.copsyc.2015.07.018

[ref123] Morgan H. O’donovan A. Almeida R. Lin A. Perry Y. (2020). The role of the avatar in gaming for trans and gender diverse young people. Int. J. Environ. Res. Public Health 17:8617. doi: 10.3390/ijerph17228617 33233536 PMC7699515

[ref124] Morgenroth T. Stratemeyer M. Paaßen B. (2020). The gendered nature and malleability of gamer stereotypes. Cyberpsychol. Behav. Soc. Netw. 23, 557–561. doi: 10.1089/cyber.2019.0577, 32486915

[ref125] Msosa S. K. (2022). The impact of corporate social investment on customer loyalty in the gaming industry. Bus. Ethics Leadership 6, 38–48. doi: 10.21272/bel.6(4).38-48.2022

[ref9012] Mulak A. Winiewski M. H. (2021). Virtual contact hypothesis: preliminary evidence for intergroup contact hypothesis in interactions with characters in video games. Cyberpsychology: Journal of Psychosocial Research on Cyberspace 15:Article 6. doi: 10.5817/CP2021-4-6

[ref126] Naidoo R. Coleman K. Guyo C. (2020). Exploring gender discursive struggles about social inclusion in an online gaming community. Inf. Technol. People 33, 576–601. doi: 10.1108/ITP-04-2019-0163

[ref9013] Nardi B. Harris J. (2006). “Strangers and friends: collaborative play in world of Warcraft” in Proceedings of the 2006 20th Anniversary Conference on Computer Supported Cooperative Work, 149–158. doi: 10.1145/1180875.1180898

[ref127] Nelson Laird T. F. (2011). Measuring the diversity inclusivity of college courses. Res. High. Educ. 52, 572–588. doi: 10.1007/s11162-010-9210-3

[ref128] Newzoo (2025). 2025 Global games market report. Available online at: https://investgame.net/wp-content/uploads/2025/09/2025_Newzoo_Free_Global_Games_Market_Report.pdf (Accessed March 4, 2026).

[ref129] Okatta C. G. Ajayi F. A. Olawale O. (2024). Enhancing organizational performance through diversity and inclusion initiatives: a meta-analysis. Int. J. Appl. Res. Soc. Sci. 6, 734–758. doi: 10.51594/ijarss.v6i4.1065

[ref130] Olson D. M. Harrell P. F. (2020). “I Don't see color”: characterizing players’ racial attitudes and experiences via an anti-Bias simulation videogame. In Proceedings of the 15th International Conference on the Foundations of Digital Games (pp. 1–4).

[ref131] Olusanya E. O. (2023). Workplace diversity, equity, inclusion. J. Bus. Divers. 23, 14–23. doi: 10.33423/jbd.v23i4.6615

[ref132] Ortiz S. M. (2019). “You can say i got desensitized to it”: how men of color cope with everyday racism in online gaming. Sociol. Perspect. 62, 572–588. doi: 10.1177/0731121419837588

[ref133] Ortiz L. (2022). Risks of the metaverse: a vrchat study case. J. Intellig. Conflict Warfare 5, 53–128. doi: 10.21810/jicw.v5i2.5041

[ref134] Paaßen B. Morgenroth T. Stratemeyer M. (2017). What is a true gamer? The male gamer stereotype and the marginalization of women in video game culture. Sex Roles 76, 421–435. doi: 10.1007/s11199-016-0678-y

[ref9015] Paavilainen J. Alha K. Korhonen H. (2016). “Review of social features in social network games” in Proceedings of DiGRA/FDG 2016 Conference.

[ref135] Passmore C. J. Birk M. V. Mandryk R. L. (2018) The privilege of immersion: racial and ethnic experiences, perceptions, and beliefs in digital gaming. In Proceedings of the 2018 CHI Conference on human Factors in Computing Systems (pp. 1–19)

[ref136] Passmore C. J. Mandryk R. L. (2020). A taxonomy of coping strategies and discriminatory stressors in digital gaming. Front. Comput. Sci. 2:40. doi: 10.3389/fcomp.2020.00040

[ref137] Pauser S. Wagner U. (2020). Judging a book by its cover: assessing the comprehensibility and perceived appearance of sign language avatars. Marketing: ZFP J. Res. Manag. 42, 48–60. doi: 10.15358/0344-1369-2020-3-48

[ref138] Pech G. P. Caspar E. A. (2024). Can a video game with a fictional minority group decrease intergroup biases towards non-fictional minorities? A social neuroscience study. Int. J. Hum. Comput. Interact. 40, 482–496. doi: 10.31234/osf.io/dfhza

[ref139] Peng W. Lin J. Pfeiffer K. A. Winn B. (2012). Need satisfaction supportive game features as motivational determinants: an experimental study of a self-determination theory guided exergame. Media Psychol. 15, 175–196. doi: 10.1080/15213269.2012.673850

[ref140] Pennington C. R. Kaye L. K. McCann J. J. (2018). Applying the multi-threat framework of stereotype threat in the context of digital gaming. PLoS One 13:e0192137. doi: 10.1371/journal.pone.0192137, 29444126 PMC5812608

[ref141] Perera C. Salamanca-Sanabria A. Caballero-Bernal J. Feldman L. Hansen M. Bird M. . (2020). No implementation without cultural adaptation: a process for culturally adapting low-intensity psychological interventions in humanitarian settings. Confl. Heal. 14:46. doi: 10.1186/s13031-020-00290-0, 32684948 PMC7362525

[ref142] Perry K. (2022). Damsels and darlings: decoding gender equality in video game communities. Feminist Media Stud. 22, 1102–1119. doi: 10.1080/14680777.2021.1883085

[ref144] Phidd N. N. (2018). A call of duty to counterstrike: cyberharassment and the toxic gaming culture plaguing female gamers and developers. J. Race Gender Soc. Just. 25:461.

[ref145] Phillips M. J. (2025). “Loading… representation”: qualitatively exploring intersecting identities in gaming culture. SN Soc. Sci. 5:58. doi: 10.1007/s43545-025-01097-7

[ref146] Pollok P. Amft A. Diener K. Lüttgens D. Piller F. T. (2021). Knowledge diversity and team creativity: how hobbyists beat professional designers in creating novel board games. Res. Policy 50:104174. doi: 10.1016/j.respol.2020.104174

[ref147] Pramono M. F. Renalda K. Warnars H. L. H. S. (2018). Matchmaking problems in MOBA games. Indones. J. Electr. Eng. Comput. Sci. 11, 908–917. doi: 10.11591/ijeecs.v11.i3.pp908-917

[ref148] Pyae A. (2018). Understanding the role of culture and cultural attributes in digital game localization. Entertain. Comput. 26, 105–116. doi: 10.1016/j.entcom.2018.02.004

[ref149] Ratan R. A. Taylor N. Hogan J. Kennedy T. Williams D. (2015). Stand by your man: an examination of gender disparity in league of legends. Games Cult. 10, 438–462. doi: 10.1177/1555412014567228

[ref150] Reza A. Chu S. Nedd A. Gardner D. (2022). Having skin in the game: how players purchase representation in games. Convergence 28, 1621–1642. doi: 10.1177/13548565221099713

[ref151] Richard G. T. Gray K. L. (2018). Gendered play, racialized reality: black cyberfeminism, inclusive communities of practice, and the intersections of learning, socialization, and resilience in online gaming. Front. J. Women Stud. 39, 112–148. doi: 10.1353/fro.2018.a690812

[ref152] Richter K. (2014). Beginning iOS Social Games. New York: Apress.

[ref153] Rieger D. Wulf T. Kneer J. Frischlich L. Bente G. (2014). The winner takes it all: the effect of in-game success and need satisfaction on mood repair and enjoyment. Comput. Human Behav. 39, 281–286. doi: 10.1016/j.chb.2014.07.037

[ref154] Robinson J. A. (2023). “I Ain’t no girl”: exploring gender stereotypes in the video game community. West. J. Commun. 87, 857–878. doi: 10.1080/10570314.2022.2130004

[ref155] Rogers E. M. (1995). Lessons for guidelines from the diffusion of innovations. Jt. Comm. J. Qual. Improv. 21, 324–328. doi: 10.1016/S1070-3241(16)30155-9, 7581733

[ref301] Rodrigues L. C. Mustaro P. N. (2007). Social network analysis of virtual communities in online games. IADIS International Journal on Computer Science and Information Systems, 3, 13–26.

[ref156] Rogers E. M. Singhal A. Quinlan M. M. (2014) "Diffusion of innovations," An Integrated Approach to Communication Theory and Research. (London: Routledge), 432–448.

[ref157] Romero M. Usart M. Ott M. Earp J. de Freitas S. Arnab S. (2012) "Learning through playing for or against each other? Promoting collaborative learning in digital game based learning," ECIS 2012 Proceedings, Paper 93.

[ref158] Saldanha L. da Marques Silva S. Ferreira P. D. (2024). Could this really be a place for me? ‘Women’s experiences in game jams and video game communities. J. Gend. Stud. 33, 431–445. doi: 10.1080/09589236.2023.2264222

[ref159] Saldanha L. Sofia Marques d. S. Ferreira P. D. (2023). “Community” in video game communities. Games Cult. 18, 1004–1022. doi: 10.1177/15554120221150058

[ref160] Sanghvi H. Bhavsar R. Hundlani V. Gohil L. Vyas T. Nair A. . (2024). MetaHate: AI-based hate speech detection for secured online gaming in metaverse using blockchain. Secur. Priv. 7:e343. doi: 10.1002/spy2.343

[ref161] Sathy V. Hogan K. A. (2019). How to Make Your Teaching More Inclusive. Washington, D.C.: The Chronicle of Higher Education. Available online at: https://www.chronicle.com/article/how-to-make-your-teaching-more-inclusive/ (Accessed April 4, 2026).

[ref162] Sato Y. (2024). “Cross-cultural game studies,” In: Lee, N. ed., Encyclopedia of Computer Graphics and Games, (Cham: Springer International Publishing), 485–490. doi: 10.1007/978-3-031-23161-2_400

[ref163] Schnepel S. Krähenmann H. Sermier Dessemontet R. Moser Opitz E. (2020). The mathematical progress of students with an intellectual disability in inclusive classrooms: results of a longitudinal study. Math. Educ. Res. J. 32, 103–119. doi: 10.1007/s13394-019-00295-w

[ref164] Shaw A. (2017). Talking to gaymers: questioning identity, community and media representation. Westminster Pap. Commun. Cult. 9:150. doi: 10.16997/wpcc.150

[ref165] Shaw A. Friesem E. (2016). Where is the queerness in games?: types of lesbian, bisexual, transgender, and queer content in games. Int. J. Commun. 10(2016) 3877–3889.

[ref166] Shelton S. A. Coogler C. H. Watson V. (2023). Queer worldmaking in animal crossing: disruptive and joyful critical media literacies. Int. J. Critical Media Literacy 3, 120–140. doi: 10.1163/25900110-03020004

[ref167] Shibuya A. Okura H. Shoun A. Asou N. (2019). Male and female game players’ preferences for game characters and real-world personalities in Japan. In Proceedings of DiGRA 2019 Conference: Game, Play and the Emerging Ludo-Mix.

[ref168] Siitonen M. (2015). Communication in video games: from players to player communities. Commun. Technol. 5:103.

[ref169] Silva J. P. N. Valadares G. C. Pedrosa G. Rezende D. C. Cappelle M. C. A. Assis F. A. A. (2023). Gender imbalance in MMORPG: the case of world of Warcraft in Brazil. Feminist Media Stud. 23, 289–305. doi: 10.1080/14680777.2021.1973060

[ref170] Singh A. Halgamuge M. N. Moses B. (2019). An analysis of demographic and behavior trends using social media: Facebook, twitter, and Instagram. Soc. Netw. Analyt. 11:87. doi: 10.1016/B978-0-12-815458-8.00005-0

[ref171] Smith A. C. Thakore B. K. (2023). Let's play, zoomers: cultural authority and stereotypical representations in video games. Sociation 22, 6–20.

[ref172] Snyder Broussard M. J. (2012). Digital games in academic libraries: a review of games and suggested best practices. Ref. Serv. Rev. 40, 75–89. doi: 10.1108/00907321211203649

[ref173] Sobieraj S. (2018). Bitch, slut, skank, cunt: patterned resistance to women’s visibility in digital publics. Inf. Commun. Soc. 21, 1700–1714. doi: 10.1080/1369118X.2017.1348535

[ref174] Srikasetsarakul A. Vungthong S. (2019). An examination of player and spectator swearing in multiplayer online gaming. Int. J. Communic. Linguist. Stud. 17:59. doi: 10.18848/2327-7882/cgp/v17i01/59-82

[ref175] Statista (2024) Number of Video Game Users Worldwide from 2019 to 2029 (in billions)

[ref176] Straits Research (2024). Gaming Market Size, Share and Trends Analysis Report by Device (Console, Mobile, Computer), by Type (Online, Offline) and by Region (North America, Europe, APAC, Middle East and Africa, LATAM) Forecasts, 2025–2033. Pune, Maharashtra, India: Straits Research.

[ref177] Sublette V. A. Mullan B. (2012). Consequences of play: a systematic review of the effects of online gaming. Int. J. Ment. Health Addict. 10, 3–23. doi: 10.1007/s11469-010-9304-3

[ref178] TaeHyuk Keum B. Hearns M. (2022). Online gaming and racism: impact on psychological distress among Black, Asian, and Latinx emerging adults. Games Cult. 17, 445–460. doi: 10.1177/15554120211039082

[ref179] Tang W. Y. Fox J. (2016). Men's harassment behavior in online video games: personality traits and game factors. Aggress. Behav. 42, 513–521. doi: 10.1002/ab.21646, 26880037

[ref180] Tang W. Y. Reer F. Quandt T. (2020). Investigating sexual harassment in online video games: how personality and context factors are related to toxic sexual behaviors against fellow players. Aggress. Behav. 46, 127–135. doi: 10.1002/ab.21873, 31736097

[ref181] Tangney J. P. Wagner P. Fletcher C. Gramzow R. (1992). Shamed into anger? The relation of shame and guilt to anger and self-reported aggression. J. Pers. Soc. Psychol. 62:669. doi: 10.1037//0022-3514.62.4.669, 1583590

[ref182] Teng C. (2017). Impact of avatar identification on online gamer loyalty: perspectives of social identity and social capital theories. Int. J. Inf. Manag. 37, 601–610. doi: 10.1016/j.ijinfomgt.2017.06.006

[ref183] Thorne S. L. Black R. W. Sykes J. M. (2009). Second language use, socialization, and learning in internet interest communities and online gaming. Mod. Lang. J. 93, 802–821. doi: 10.1111/j.1540-4781.2009.00974.x

[ref305] Thiel S. K. Lyle P. (2019). Malleable games: A literature review on communities of game modders. In Proceedings of the 9th International Conference on Communities & Technologies – Transforming Communities 198–209. doi: 10.1145/3328320.3328393

[ref184] Trudgett-Klose L. H. McLinton S. S. (2024). “Progamers” & cyberbullying: workplace bullying & sexual harassment in professional video gaming. Entertain. Comput. 50:100702. doi: 10.1016/j.entcom.2024.100702

[ref185] Tschang F. T. (2007). Balancing the tensions between rationalization and creativity in the video games industry. Organ. Sci. 18, 989–1005. doi: 10.1287/orsc.1070.0299

[ref186] Tushya Chhabra D. Abraham B. (2023). Social networking or social isolation? A systematic review on socio-relational outcomes for members of online gaming communities. Games Cult. 20:15554120231201760. doi: 10.1177/15554120231201760

[ref302] Two Lonely Penguins. (2025). For honor – most toxic player ever! (with angry voice chat) [Video]. YouTube. Available at: https://www.youtube.com/watch?v=80hE3FMSWuE

[ref187] Vaccaro A. August G. Kennedy M. S. (2011). Safe Spaces: Making Schools and Communities Welcoming to LGBT Youth. New York, NY, USA: Bloomsbury Publishing USA.

[ref188] Vajawat B. Varshney P. Banerjee D. (2021). Digital gaming interventions in psychiatry: evidence, applications and challenges. Psychiatry Res. 295:113585. doi: 10.1016/j.psychres.2020.113585, 33303223

[ref189] Valentine K. D. Jensen L. J. (2016). Examining the Evolution of Gaming and its Impact on Social, Cultural, and Political Perspectives. Hershey PA, USA: IGI Global.

[ref190] Van der Velden M. Mörtberg C. (2015). “Participatory design and design for values,” in van den Hoven, J., Vermaas, P., van de Poel, I. (eds)., Handbook of Ethics, Values, and Technological Design: Sources, Theory, Values and Application Domains, (Dordrecht: Springer), 41–66. doi: 10.1007/978-94-007-6994-6_33-1

[ref191] Vang M. H. Fox J. (2014). Race in virtual environments: competitive versus cooperative games with black or white avatars. Cyberpsychol. Behav. Soc. Netw. 17, 235–240. doi: 10.1089/cyber.2013.0289, 24152253

[ref192] Vergel P. La parra-Casado D. Vives-Cases C. (2024). Examining cybersexism in online gaming communities: a scoping review. Trauma Violence Abuse 25, 1201–1218. doi: 10.1177/15248380231176059, 37243440

[ref303] Villar A. León C. (2022). Improving performance in collaborative games through personality-based matchmaking. CEUR Workshop Proceedings.

[ref304] Vicencio-Moreira R. Mandryk R. L. Gutwin C. (2015). Now you can compete with anyone: Balancing players of different skill levels in a first-person shooter game. In Proceedings of the 33rd Annual ACM Conference on Human Factors in Computing Systems. 2255–2264. doi: 10.1145/2702123.2702242, 37243440

[ref193] Voida A. Carpendale S. Greenberg S. (2009) The MII and the WII: Emphasizing the Individual and the Group in Console Gaming. Research Report 2009: 931-10, Department of Computer Science, University of Calgary, Calgary, Alberta T2N 1N4, Canada. Available online at: https://ucalgary.scholaris.ca/server/api/core/bitstreams/54874877-c323-4104-980e-3d8c325d953a/content (Accessed April 4, 2026).

[ref194] Waechter N. Meschik M. (2023). Peer socialization of male adolescents in digital games: achievement, competition, and harassment. Communications 48, 457–481. doi: 10.1515/commun-2021-0079

[ref195] Weber R. Behr K. DeMartino C. (2014). Measuring interactivity in video games. Commun. Methods Meas. 8, 79–115. doi: 10.1080/19312458.2013.873778

[ref196] Weigel S. Rudnick J. (2023). The use and importance of gaming and roleplay in identity negotiation. Commun. Theater Assoc. Minn. J. 46:8. doi: 10.56816/2471-0032.1161

[ref197] Wells G. Romhányi Á. Steinkuehler C. (2025). Hate speech and hate-based harassment in online games. Front. Psychol. 15:1422422. doi: 10.3389/fpsyg.2024.1422422, 40046807 PMC11881651

[ref198] Westin T. Furöstam M. Yasasindhu R. Norberg L. Wiklund M. Mozelius P. (2015). “Balancing game universes for playing without sight or hearing,” in Cecilia Sik-Lányi, Evert-Jan Hoogerwerf, Klaus Miesenberger, Peter Cudd (Ed.), Assistive technology: Building bridges. Paper presented at AAATE 2015, Budapest, Hungary, September 9-12, 2015, (Amsterdam, Netherlands: IOS Press), 372–377. 26294500

[ref199] Wijkstra M. Rogers K. Mandryk R. L. Veltkamp R. C. Frommel J. (2024). How to tame a toxic player? A systematic literature review on intervention systems for toxic behaviors in online video games. Proc. ACM Hum. Comput. Interact. 8, 1–32. doi: 10.1145/367708039286336

[ref200] Williams D. (2006). Why game studies now? Gamers don't bowl alone. Games Cult. 1, 13–16. doi: 10.1177/1555412005281774

[ref201] Wong S. Ratan R. (2024). How does exposure to general and sexual harassment relate to female gamers’ coping strategies and mental health? Games Cult. 19, 670–689. doi: 10.1177/15554120231177600

[ref202] Woodhouse T. N. (2022). Digital archives, fandom histories, and the reproduction of the hegemony of play. Transform. Works Cult. Estados Unidos América 37:2105. doi: 10.3983/twc.2022.2105

[ref203] Wu A. M. Lai M. H. Yu S. (2017). Psychological need satisfaction, gaming motives, and internet gaming disorder. Eur. Health Psychol. 19:1189.

[ref204] Yao S. X. Ellithorpe M. E. Ewoldsen D. R. Boster F. J. (2023). Development and validation of the female gamer stereotypes scale. Psychol. Pop. Media 12:393. doi: 10.1037/ppm0000430

[ref205] Yin M. Qiu B. He X. Tao Z. Zhuang C. Xie Q. . (2022). Effects of reward and punishment in prosocial video games on attentional bias and prosocial behaviors. Comput. Hum. Behav. 137:107441. doi: 10.1016/j.chb.2022.107441

[ref206] Zhang Z. Mu H. Huang S. (2023). Playing to save sisters: how female gaming communities foster social support within different cultural contexts. J. Broadcast. Electron. Media 67, 693–713. doi: 10.1080/08838151.2023.2254432

[ref207] Zhou Y. Peterson Z. D. (2025). Women's experiences of sexual harassment in online gaming. Violence Against Women 31, 2037–2052. doi: 10.1177/10778012241252021, 38712842

[ref208] Zorlu D. Özkan N. (2020). Women on twitch Turkey: affective communities and female solidarity under patriarchy and postfeminism. Gender Forum 19, 105–127. doi: 10.18716/ojs/gefo/2020.2532

[ref209] Zubiri-Esnaola H. Vidu A. Rios-Gonzalez O. Morla-Folch T. (2020). Inclusivity, participation and collaboration: learning in interactive groups. Educ. Res. 62, 162–180. doi: 10.1080/00131881.2020.1755605

