# Supplementary material for: Inclusion and exclusion in gaming communities: a systematic review and future directions to increase inclusivity
Source: Front Psychol. 2026 May 1;17:1796305. doi: 10.3389/fpsyg.2026.1796305 (PMC13176318; doi:10.3389/fpsyg.2026.1796305)
Supplement: Supplementary file 1 [file Supplementary_file_1.docx]

Supplementary Material

# Definitions and Conceptualizations

## Gaming, Community, and Culture

The term "gaming" covers a wide range of activities centered on play, typically structured by rules and undertaken for enjoyment, entertainment, or competition. This includes both traditional forms, such as board games and sports, and the increasingly common digital games played on computers, consoles, and mobile devices (Mäyrä, 2015; Stenros, Paavilainen, & Mäyrä, 2011). Currently, "gaming" is predominantly associated with video gaming, defined by Dictionary.com as "the playing of video or computer games” (n.d).

The concept of "community" has been extensively explored across various disciplines, leading to diverse definitions but also converging on key themes. Communities are generally viewed as groups of individuals who interact and support each other, sharing experiences, characteristics, or a sense of belonging (Cobigo, Martin, & Mcheimech, 2016). Gaming communities embody the convergence of these concepts. They form social networks around shared interests in gaming activities or specific games, offering spaces for collaboration, socialization, and interactions that often go beyond the game itself (Mäyrä, 2015). These communities vary, and may be closely knit, with strong social bonds and obligations, or loosely connected, serving primarily functional purposes (Brint, 2001; Ducheneaut et al., 2006). They also differ greatly in structure, size, and purpose, accommodating various gaming preferences, platforms, and demographics, and while some communities are highly competitive and focused on achievement, others emphasize social interaction and creative expression (Mäyrä, 2015).

While offline communities are often linked to physical proximity, contemporary perspectives recognize the emergence of communities not limited by geography, such as online communities (Armstrong & Hagel, 2009). This difference allows online communities to flourish, as the sense of belonging and shared interests can transcend geographical boundaries (Baker & Ward, 2002; Gochenour, 2006). Tönnies (1957) emphasized that one crucial distinction between communities and societies lies in the way social order is maintained. Whereas societies rely on more formal mechanisms like laws and institutions to maintain order, communities foster a strong sense of belonging that creates an informal social order based on shared values and traditions--i.e., the communtiey’s culture.

The culture of gaming communities encompasses the shared norms, values, practices, and identities that arise from social interactions and influence the gaming experience. This culture can be seen in the development of specific languages and slang, social conventions and expectations around gameplay and player interaction, and the broader practices and discourses around games, such as content creation and forum discussions (Consalvo, 2007; Mäyrä, 2008; Jenkins, 2006). Thus, the culture within these communities is both formed by and informs interactions, establishing norms and practices. Members of these communities are often characterized by their level of involvement and the extent to which they embrace and influence the community's culture (Mäyrä, 2015).

Most of the reviewed studies either focus on a specific community or analyze individual gamers to infer the culture of gaming communities as a whole. Consequently, we often discuss our findings in terms of the broader gaming community culture and note when a study specifically addresses a particular gaming community.

## Social Exclusion

Social exclusion refers to the practices and processes that block or deny certain individuals and groups from full access to rights, opportunities, and resources that are normally available to others within a society (Byrne, 2005; Chakravarty & D'Ambrosio, 2006). Social exclusion can manifest through various forms of disadvantage, marginalization, and relegation to the fringes of society based on characteristics such as social class, race, gender, or other identities (Silver, 1994). For example, language and discourses can keep the dominant culture, thus further maintaining exclusion (Chakravarty & D'Ambrosio, 2006). In the context of gaming communities, social exclusion manifests through marginalization and underrepresentation of players based on factors like gender, race, sexuality, age, or disability. This exclusion can take various forms, including stereotypical or problematic portrayals in-game, harassment and discrimination within gaming spaces, and barriers that limit full participation and access for marginalized groups (Gray & Leonard, 2018; Grammenos et al., 2009).

Social exclusion is a significant barrier to achieving inclusion and diversity within various contexts, including gaming communities. By understanding the factors and processes that contribute to social exclusion, we can better identify strategies for promoting inclusion and creating more equitable and diverse gaming spaces. In the current review, we will describe and analyze the various practices and manifestations of social exclusion within gaming communities cultures, as identified in the referenced studies.

# References:

Armstrong, A., & Hagel, J. (2009). The real value of online communities. In *Knowledge and communities* (pp. 85-95). Routledge.

Baker, P. M., & Ward, A. C. (2002). Bridging temporal and spatial" gaps": The role of information and communication technologies in defining communities. *Information, Communication & Society*, *5*(2), 207-224.

Brint, S. (2001). Gemeinschaft revisited: A critique and reconstruction of the community concept. *Sociological theory*, *19*(1), 1-23.

Byrne, D. (2005). *Social exclusion*. McGraw-Hill Education (UK).

Chakravarty, S. R., & D'Ambrosio, C. (2006). The measurement of social exclusion. *Review of Income and wealth*, *52*(3), 377-398.

Cobigo, V., Martin, L., & Mcheimech, R. (2016). Understanding community. *Canadian Journal of Disability Studies*, *5*(4), 181-203.

Consalvo, M. (2009). There is no magic circle. *Games and culture*, *4*(4), 408-417.

Dictionary.com. (n.d.) Video Gaming. In *Dictionary.com dictionary*. Retrieved July 09, 2025, from <https://www.dictionary.com/browse/video-gaming>.

Ducheneaut, N., Yee, N., Nickell, E., & Moore, R. J. (2006, April). " Alone together?" Exploring the social dynamics of massively multiplayer online games. In *Proceedings of the SIGCHI conference on Human Factors in computing systems* (pp. 407-416).

Gochenour, P. H. (2006). Distributed communities and nodal subjects. *New Media & Society*, *8*(1), 33-51.

Grammenos, D., Savidis, A., & Stephanidis, C. (2009). Designing universally accessible games. *Computers in Entertainment (CIE)*, *7*(1), 1-29.

Gray, K. L., & Leonard, D. J. (Eds.). (2018). *Woke gaming: Digital challenges to oppression and social injustice*. University of Washington Press.

Jenkins, H. (2006). *Fans, bloggers, and gamers: Exploring participatory culture*. nyu Press.

Mäyrä, F. (2008). *An introduction to game studies*. Sage.

Mäyrä, F. (2015). Mobile games. *The international encyclopedia of digital communication and society*, *3*, 610-614.

Silver, H. (1994). Social exclusion and social solidarity: Three paradigms. *Int'l Lab. Rev.*, *133*, 531.

Stenros, J., Paavilainen, J., & Mäyrä, F. (2011). Social interaction in games. *International Journal of arts and technology*, *4*(3), 342-358.

Tönnies, F. (1957). *Community and society.* [Gemeinschaft und Gesellschaft]. Michigan State Univer. Press.
